# Genome-wide identification and expression analysis of the ZF-HD gene family in pea (*Pisum sativum* L.)

**DOI:** 10.3389/fgene.2022.1089375

**Published:** 2023-01-05

**Authors:** Bowen Shi, Inzamam Ul Haq, Sajid Fiaz, Badr Alharthi, Ming-Long Xu, Jian-Lin Wang, Wei-Hai Hou, Xi-Bo Feng

**Affiliations:** ^1^ Plant Sciences College, Tibet Agricultural and Animal Husbandry University, Linzhi, Tibet, China; ^2^ College of Plant Protection, Gansu Agricultural University, Lanzhou, Gansu, China; ^3^ Department of Plant Breeding and Genetics, The University of Haripur, Haripur, Pakistan; ^4^ Department of Biology, University College of Al Khurmah, Taif University, Saudi Arabia

**Keywords:** transcription factors, ZF-HD proteins, low-temperature stress, biological functions, gene function

## Abstract

Pea is a conventional grain-feed-grass crop in Tibet and the only high-protein legume in the region; therefore, it plays an important role in Tibetan food and grass security. Zinc finger-homeodomain (ZF-HD) belongs to a family of homozygous heterotypic cassette genes, which play an important role in plant growth, development, and response to adversity stress. Using a bioinformatics approach, 18 PsZF-HD family members were identified. These genes were distributed across seven chromosomes and two scaffold fragments, and evolutionary analysis classified them into two subgroups, MIF and ZHD. The MIF subgroup was subdivided into three subclasses (PsMIFⅠ–III), and the ZHD subgroup was subdivided into five subclasses (ZHDⅠ–V). The PsZF-HD members were named PsMIF1–PsMIF4 and PsZHD1–PsZHD14. Twelve conserved motifs and four conserved domains were identified from PsZF-HD family, of which MIF subgroup only contained one domain, while ZHD subgroup contained two types of domains. In addition, there were significant differences in the three-dimensional structures of the protein members of the two subgroups. Most PsZF-HD genes had no introns (13/18), and only five genes had one intron. Forty-five cis-acting elements were predicted and screened, involving four categories: light response, stress, hormone, and growth and development. Transcriptome analysis of different tissues during pea growth and development showed that *PsZHD*11, 8, 13, 14 and MIF4 were not expressed or were individually expressed in low amounts in the tissues, while the other 13 *PsZF-HD*s genes were differentially expressed and showed tissue preference, as seen in aboveground reproductive organs, where *PsZHD*6, 2, 10 and *MIF*1 (except immature seeds) were highly expressed. In the aerial vegetative organs, *PsZHD*6, 1, and 10 were significantly overexpressed, while in the underground root system, *PsMIF*3 was specifically overexpressed. The leaf transcriptome under a low-nitrogen environment showed that the expression levels of 17 *PsZF-HD*s members were upregulated in shoot organs. The leaf transcriptome analysis under a low-temperature environment showed stress-induced upregulation of *PsZHD*10 and one genes and down-regulation of *PsZHD*6 gene. These results laid the foundation for deeper exploration of the functions of the *PsZF-HD* genes and also improved the reference for molecular breeding for stress resistance in peas.

## 1 Introduction

Pea (*Pisum sativum* L.) is a conventional grain-feed-grass crop that has been grown for a long time in the cold and dry Qinghai-Tibet Plateau and is a major crop for abandoned relief. It has a wide range of distribution and shows great resistance to cold, drought, and dryness. Pea can grow up to 4,700 m above the sea level, and it is often mixed with highland staple crops, such as hulless barley and oilseed rape. Plants with strong resistance against stress can adapt to the severe cold and drought environment of the plateau. Transcription factors (TFs) initiate target gene expression by binding to specific cis-acting elements in the promoter regions of related genes to regulate plant growth and development and resistance to stress (Zhou et al., 2019). Zinc finger homeodomain (ZF-HD) proteins are plant-specific TFs that play important roles in the regulation of flower development as well as biotic and abiotic stresses (Windhövel et al., 2001; Lai et al., 2021). ZF-HD proteins are classified into two subfamilies, the Zinc Finger Homotypic Box (ZHD) and the Mini Zinc Finger (MIF), according to a phylogenetic tree (Hu et al., 2008). Three *MIF* genes were identified for the first time in *Arabidopsis thaliana*, encoding proteins similar to the ZF structural domain of ZF-HD proteins but lacking the HD structural domain (Hu and Ma, 2006). To date, the origin and evolutionary relationship between the two remain unclear, but both belong to the ZF-HD family. The distinctive structural features of ZHDs comprise a zinc finger (ZF) structural domain at the N-terminal and a conserved homologous heterotetrameric box HD (homeo-domain) at the C-terminal (Wang et al., 2016). ZFs contain zinc ions and five conserved cysteine residues or at least three conserved histidine residues (Krishna et al., 2003). ZFs are widely present in different regulatory proteins, bind specifically to DNA/RNA sequences and enhance protein–DNA interactions, and are mediated by the HD structural domain (Xie et al., 2019). HD encodes a highly conserved DNA structural domain consisting of 60 amino acids that fold to form a 3-helix structure, with the first and second helix forming a loop between them and the second and third helix regions forming a helix-turned-helical structure (Mukherjee et al., 2009). Most HD proteins specifically adsorb to the major groove of DNA to activate and repress the expression of target genes (Mukherjee et al., 2009; Hu et al., 2018). Based on the HD amino acid sequence and other conserved motifs accompanying it, HD-bearing proteins are classified into typical HD structural domains (with 60 amino acids in length) and atypical HD structural domains (with variation in amino acid length). The latter is known as three amino acid loop extension homozygous heterotypic box superfamily proteins, encoding 63 with three additional amino acid residues (P-Y-P) between the first and second helices (Chen et al., 2003). In rice, 107 HD proteins were identified and classified into 10 subfamilies, including ZF-HD, KNOX I, KNOX II, WOX, HD-Zip I, BLH, HD-Zip II, HD-Zip III, HD-Zip IV, and PHD, based on the sequence length, structure, HD position, and other structural domains containing HD proteins (Jain et al., 2008). Subsequently, Mukherjee (2009) systematically studied plant homozygous heterotypic cassette genes and classified them into 14 subfamilies with the addition of NDX, DDT, PHD, LD, SAWADEE, and PINTOX (Mukherjee et al., 2009). ZF-HD proteins often bind to ATTA elements of DNA sequences to form homodimers and heterodimers (Tan and Irish, 2006).

ZF structures are widely found in a variety of regulatory proteins and play an important role in the formation of homodimers or heterodimers of different members of the ZF-HD family (Windhövel et al., 2001). A typical ZF structure contains two pairs of conserved cysteine residues or histidine residues and is coordinated to a single zinc ion to form a finger-like loop (Klug and Schwabe, 1995). They are classified into different types according to the number and nature of residues bound to zinc ions and zinc-binding protein residues. For example, C2H2, C3H, and C2C2 ZFs interact with one zinc ion, while PHD and LIM ZFs can interact with two zinc ions, with the C2H2 type being the most common (Englbrecht et al., 2004; Yanagisawa, 2004). Single proteins can have one or more ZF structures, and ZFs can recognize and bind DNA, RNA, DNA-RNA double-stranded molecules, or proteins (Takatsuji, 1999; Krishna et al., 2003) and regulate gene expression at the transcriptional and translational levels, which can play an important role in plant stress response and defense activation (Mackay and Crossley, 1998). In ZF-HD proteins, HD binds to DNA, and the ZF domain enhances HD-mediated protein–DNA interactions (Windhövel et al., 2001; Hu et al., 2008).

Currently, the ZF-HD family is widely studied in several higher plants, including 17 members in *Arabidopsis thaliana* (Hu et al., 2008), 32 members in tobacco (Niu et al., 2021; Sun et al., 2021), 37 members in wheat (Liu et al., 2021), 13 members in cucumber (Lai et al., 2021), 24 members in maize (Jing et al., 2022), 18 members in tea tree (Zhou et al., 2021), 60 members in alfalfa (He et al., 2022), 20 members in buckwheat, 62 members in kale type oilseed rape (Song et al., 2019), and 31 members in Chinese cabbage (Wang et al., 2016). To date, the biological functions of most of the *ZF-HD* genes identified in *Arabidopsis thaliana* have been characterized as being involved in blue light signaling regulation, vascular bundle development, outer cell biosynthesis of organs, stress response to adversity, and anthocyanin synthesis. For example, multiple *ZF-HD*s in *Arabidopsis thaliana* are involved in the regulation of floral organ development and functional sink residues, and similar findings have been reported in barley and wheat (Tran et al., 2007; Abu-Romman and Al-Hadid, 2017). Drought, salinity, and external application of abscisic acid induce *AtZHD*1 expression (Wang et al., 2014). The overexpression of *ZF-HD*1 and *NAC* genes enhances drought resistance in *Arabidopsis thaliana* (Tran et al., 2007; Hu et al., 2008). Using *Arabidopsis thaliana* overexpressing *MIF*1, it has been demonstrated that *MIF*1 regulates multiple hormones and affects plant growth and development. In addition, *MIF*1 overexpression impedes ZHD protein functions, and this may result from interactions with the ZF structural domain (Hu and Ma, 2006). *ZF-HD*4 expression can be induced by drought and salt stress (Hu et al., 2008). It has also been reported that the overexpression of *ZF-HD*5 can result into large plant leaves (Hong et al., 2011). *ZF-HD*10 is highly expressed in the hypocotyl and induces the expression of hypocotyl elongation-related genes *HFR*1 (Long hypocotyl in far-red) and *ATXTH*17 (Xyloglucan endotransglucosylase/hydrolase) (Shalmani et al., 2019). *ZF-HD*8 is highly expressed in floral organs and plays an important role in flower development (Hu et al., 2008). It has also been shown that most *ZF-HD* genes are expressed in floral organs, significantly associated with flowering-related hormones (GA, 6-BA), and may be involved in the regulation of floral organ development (Tan and Irish, 2006; Shalmani et al., 2019). Under low temperatures, drought, and mechanical damage, all four *ZF-HD*s bind to the *DREB*1*B* promoter to activate its expression (Figueiredo et al., 2012). Soybean *ZF-HD*1 and *ZF-HD*2 are transiently heterologously expressed in *Arabidopsis thaliana* protoplasts, confirming their response to pathogen infestation and activation of *CaM4* gene expression (Park et al., 2007), which is involved in plant defense responses. In other species, *ZF-HD,* such as tomato SlZF-HD7 and buckwheat FtZF-HD1, plays an important role in leaf and flower bud development (Khatun et al., 2017; Liu et al., 2019). The expression analysis of 24 *ZF-HD* genes in maize under ABA, alpine, and drought stresses has showed that both *ZF-HD*11 and *ZF-HD*12 exhibit significant responses to abiotic stresses (Jing et al., 2022).

Pea is important for food security and the healthy development of animal husbandry in Tibet. To date, there have been no reports of systematic studies on the pea *ZF-HD* gene family. Moreover, since the pea whole genome sequencing results were published in 2022 (Yang et al., 2022), recent bioinformatics advances have made it easy to analyze the pea ZF-HD family variation on a broad genomic scale. Although the ZF-HD gene family has been identified in several species, the effects of its members on the growth, development, yield, and quality of pea have not been reported. In this study, bioinformatics was used to identify the *ZF-HD* gene family based on the sequencing results of the pea genome. Its protein structure, basic physicochemical characteristics, cis-acting elements, and gene covariance were analyzed to lay the foundation for further understanding of the biological functions of the ZF-HD gene family.

## 2 Materials and methods

### 2.1 Identification of the ZF-HD gene family in pea

Genomes, protein sequences, and gff3 annotation files of dicotyledons crops pea (*Pisum sativum*), soybean (*Glycine_*max *L.*), tomato (*Solanum lycopersicum* L), cabbage (*Brassica Rapa* L.), and monocotyledonous crops like *Arabidopsis thaliana* (*Arabidopsis thaliana* L.), rice (*Oryza sativa L.*) were obtained from Ensembl plants (http://plants.ensembl.org/index.html) and Phytozome (https://phytozome-next.jgi.doe.gov/) public databases. Pea ZF-HD family members were searched and identified by two methods. Firstly, a BLAST search of the pea proteome database was performed using the identified AtZF-HD protein sequence as a probe to obtain the first candidate ZR-HD family members. Second, the Pfam number (PF04770) of the ZF-HD gene family structural domain was searched on the Pfam website(http://pfam-legacy.xfam.org/), the corresponding Hidden Markov Model profile (HMM) was downloaded, the protein sequences containing similar structural domains to the Hidden Markov Model (E-value <1.2e-^28^) were searched for the first time with the HMMER software(version 3.3.2), and the ZF-HD structural domain sequences were extracted, and the ZF-HD structural domain sequences were analyzed with the Clustalw software(version 2.1) for multiple sequence alignment of ZF-HD structural domain sequences to construct a pea-specific stealth Markov model, search again for candidate ZF-HD family genes containing ZF-HD structural domains (E-value <.001), remove duplicate transcripts, select the longest transcripts, extract the protein sequences corresponding to the transcripts, and submit them to CDD (https:// www.ncbi.nlm.nih.gov/cdd),SMART (http://smart.embl.de/), and PFAM (http://pfam.xfam.org/) databases to confirm the structural transgressions and remove sequences that do not contain the ZF-HD structural domain. The candidate genes identified first time were compared and analyzed with those identified the second time. The full lengths of the proteins and corresponding CDS sequences of the 18 ZF-HD gene family members were finally obtained. The amino acid length, molecular weight, and isoelectric point of the 18 ZF-HD family member proteins were predicted using the online tool ExPASy-ProtParam (https://web.expasy.org/protparam/). The obtained protein sequences were analyzed for subcellular localization using the WoLF PSORT online tool(https://wolfpsort.hgc.jp/) to construct phylogenies.

### 2.2 Phylogenetic tree analysis

The full-length sequences of ZF-HD proteins from Chinese cabbage(*Brassica rapa L.*), tomato(*Solanum lycopersicum L.*), soybean(*Glycine* max *L.*), rice(*Oryza sativa L.*), and *Arabidopsis thaliana* (*Arabidopsis thaliana L.*) were compared with Clustal X1.8 multiplex using MEGA X software(Kumar et al., 2018). The comparison results were used to generate evolutionary trees using the maximum likelihood method (ML) with parameters set to bootstrap 1,000 and the model set to JTT + G (Supplementary File S2). The evolutionary tree was embellished with the online tool Evolview (https://www.evolgenius.info/evolview/) (He et al., 2016).

### 2.3 Chromosome localization, gene duplication and ka/ks analysis

Information on the location of ZF-HD family genes on chromosomes was obtained from pea genome annotation files, and their gene lengths were obtained using samtools software. Centipede maps of genes on chromosomes were drawn using the online software MG2c (http://mg2c.iask.in/mg2c_v2.1/). MCScanX software is used for analyze gene doubling and gene tandem duplication events in pea genome, Then, KaKs_Calculator software calculates ka/ks values of duplicated genes and tandem repeats.

### 2.4 Conserved structural domains, conserved motifs, and gene structure and 3D structure analysis

Online program MEME (https://meme-suite.org/meme/tools/meme) discovers novel, ***ungapped*** motifs (recurring, fixed-length patterns) in your sequences (sample output from sequences). MEME splits variable-length patterns into two or more separate motifs. MEME tools was applied to predict the conserved motifs of 18 ZF-HD family protein, setting the search to a maximum of 12 motifs with amino acid motifs ranging from 6 to 100. The exon, CDS, and UTR position information of ZF-HD family genes were extracted based on the pea genome annotation file to map their gene structures. 18 PsZF-HD protein sequences were submitted to the online software SWISS-MODEL (https://swissmodel.expasy.org), retrieved the most similar models from the PDB library to predict the three-dimensional structure of pea PsZF-HD protein.

### 2.5 Prediction of cis-acting elements in promoter regions

The online software Plant CARE (http://bioinformatics.psb.ugent.be/webtools/plantcare/html/) was used to predict the cis-acting element of the 1,500 bp sequence upstream of the ZF-HD family gene.

### 2.6 Inter-tissue differential expression analysis of PsZF-HD family genes

Transcriptome *FASTQ*data from four groups of different treatments of pea were downloaded from the NCBI SRA database (http://www.Ncbi.Nlm.Nih.gov) and the EBI-ENA database (https://www.ebi.ac.uk/ena/browser/home) for data analysis. Among them, the participating varieties in group 1 were the Kaspa variety (semi-leafless medium-high, spherical brown medium grain) and Parafield variety (common plant phenotype, spherical brown large grain) varieties with transcriptome data accession numbers PRJNA277074 and PRJNA277076, respectively (Sudheesh et al., 2015). The test items were true leaves, stipules, stems, tendrils, roots, and root tip tissues of 4-week-old seedlings, stamens at fully open flowers (10–14 days after flowering), pistils, immature pods, immature seeds (20–25 days after flowering) and rhizome nodules (3-month-old plants) and seedlings (7-day-old seedlings) collected at multiple stem nodes at different developmental stages. The second group of participating materials was two varieties of vegetable peas and grain peas, sampled at five periods of post-flowering pod development to determine the transcriptome (Yang et al., 2019). The third group of participant test materials, two pea varieties with different cold effects, a cold-resistant winter forage variety and a cold-sensitive spring dry pea variety, were subjected to low-temperature treatment at different developmental stages with transcriptome data registration number PRJNA543764(Bahrman et al., 2019). The transcriptome of the fourth group of participant materials in different N-treated peas at three developmental stages in different tissues (Alves-Carvalho et al., 2015). For the above FASTQ data according to the type of library construction, they were de-spliced with fastp software (Chen et al., 2018), and the low-quality sequences were removed according to the default parameters to obtain clean and high-quality sequences. Subsequently, they were mapping to the pea reference genome using Hisat2 software (Kim et al., 2015) to generate Sam files and converted to Bam files, and the resultant files were subjected to quality control analysis using RSeQC comparison to detect whether the sequences were normal, genome coverage, and RNA explained. Gene expression was analyzed by FPKM using Htseq-count software (Anders et al., 2015).

## 3 Results

### 3.1 Identification of the ZF-HD gene family

To identify the *ZF-HD* genes in *Pisum sativum*, two HMM analyses were performed, generating 18 PsZF*-HD* genes after confirming ZF-HD domain by SMART and NCBI Conserved Domain Search Service Supplementary File S1). The longest variable splicing was adopted for the study (Supplementary File S2). Based on the evolutionary tree and the order and position of the ZF-HD proteins on the chromosome, they were named MIF1-4 and PsZFD1-14 (Table 1). Eighteen ZF-HDs proteins had amino acid lengths in the range of 75–417 amino acids, molecular weights in the range of 46,209.52–8,126.16 Da, and isoelectric points PI in the range of 4.78–9.08. PsZHD13 had the longest amino acid sequence, the largest molecular weight, and the smallest PI value. PsMIF2 had the shortest amino acid sequence, the smallest molecular weight, and a PI value of 8.97. PsZHD5 and PsMIF4 had equal and largest PI values, both at 9.08. The subcellular localization of the PsZF-HD family showed that PsMIF1, PsZHD8, PsZHD11, and PsZHD11 were located in the cytoplasm. PsZHD10 was localized in the cytoplasm or nucleus, and PsMIF2 and PsMIF3 were localized in the chloroplast. Other 11 PsZF-HD family members were localized in the nucleus. These findings showed that the 18 psZF-HD proteins differed significantly in their sequences and characteristics.

**TABLE 1:** Detailed information for the ZF-HD gene family in *Pisum sativum.*

Gene name	Gene id	Chromosomal position	gDNA length/bp	CDS length/bp	Length/aa	PI	MW/Da	Subcellular localization	Ortholog	E-Value
*PsMIF*1	Psat1g073200.1	chr1LG6	2,169	255	84	6.81	9,250.29	cytoplasm	AT3G28917.1(*ATMIF*2)	7.3E^−33^
*PsZHD*1	Psat1g073320.1	chr1LG6	3,443	1,005	334	7.74	36,658.01	nucleus	AT5G15210.1 (*AtZHD*8)	1.5E^−71^
*PsZHD*2	Psat1g124840.1	chr1LG6	1,263	540	179	8.16	19,743.9	nucleus	—	—
*PsZHD*3	Psat2g066960.1	chr2LG1	2,451	816	271	6.41	29,669.55	nucleus	—	—
*PsZHD*4	Psat2g081040.1	chr2LG1	1914	690	229	6.59	25,188.95	nucleus	AT5G65410.1(*AtZHD*1)	3.3E^−62^
*PsZHD*5	Psat2g158960.1	chr2LG1	1,211	582	193	9.08	22,021.71	nucleus	AT2G18350.1(*AtZHD*6)	1.1E^−57^
*PsZHD*6	Psat3g198320.1	chr3LG5	2,651	1,056	351	8.15	39,379.84	nucleus	AT3G28920.1 (*AtZHD*9)	1.3E^−68^
*PsZHD*7	Psat4g001440.1	chr4LG4	1,697	1,077	358	7.26	40,133.64	nucleus	AT2G02540.1 (*AtZHD*3)	4.3E^−68^
*PsMIF*2	Psat4g050800.1	chr4LG4	1,602	228	75	8.97	8,126.16	chloroplast	AT1G74660.1 (*ATMIF*1)	4.5E^−22^
*PsZHD*8	Psat4g115280.1	chr4LG4	846	669	222	8.29	25,140.63	cytoplasm	—	
*PsZHD*9	Psat4g141240.1	chr4LG4	1,594	897	298	8.61	32,418.12	nucleus	AT1G69600.1(*AtZHD*11)	1.5E^−48^
*PsZHD*10	Psat5g045800.1	chr5LG3	2,364	813	270	5.93	29,777.07	nucleus/cytoplasm	AT4G24660.1(*AtZHD*2)	1.6E^−67^
*PsMIF*3	Psat5g176320.1	chr5LG	276	276	84	6.81	9,250.29	chloroplast	AT1G18835.1(*MIF*3)	8.7E^−42^
*PsZHD*11	Psat6g112080.1	chr6LG2	558	558	185	8.74	21,372.62	cytoplasm	AT1G14440.1(*AtZHD*4)	3.3E^−60^
*PsZHD*12	Psat7g232440.1	chr7LG7	897	693	230	8.98	26,596.48	Extracellular	—	—
*PsMIF*4	Psat0s667g0040.1	scaffold00667	3,367	302	230	9.08	25,499.03	cytoplasm	—	—
*PsZHD*13	Psat0s3255g0040.1	scaffold03255	1,254	1,254	417	4.78	46,209.52	nucleus	—	—
*PsZHD*14	Psat0s3255g0080.1	scaffold03255	1,236	603	200	6.59	21,343.39	nucleus	—	—

### 3.2 Phylogenetic tree analysis

**FIGURE 1:**
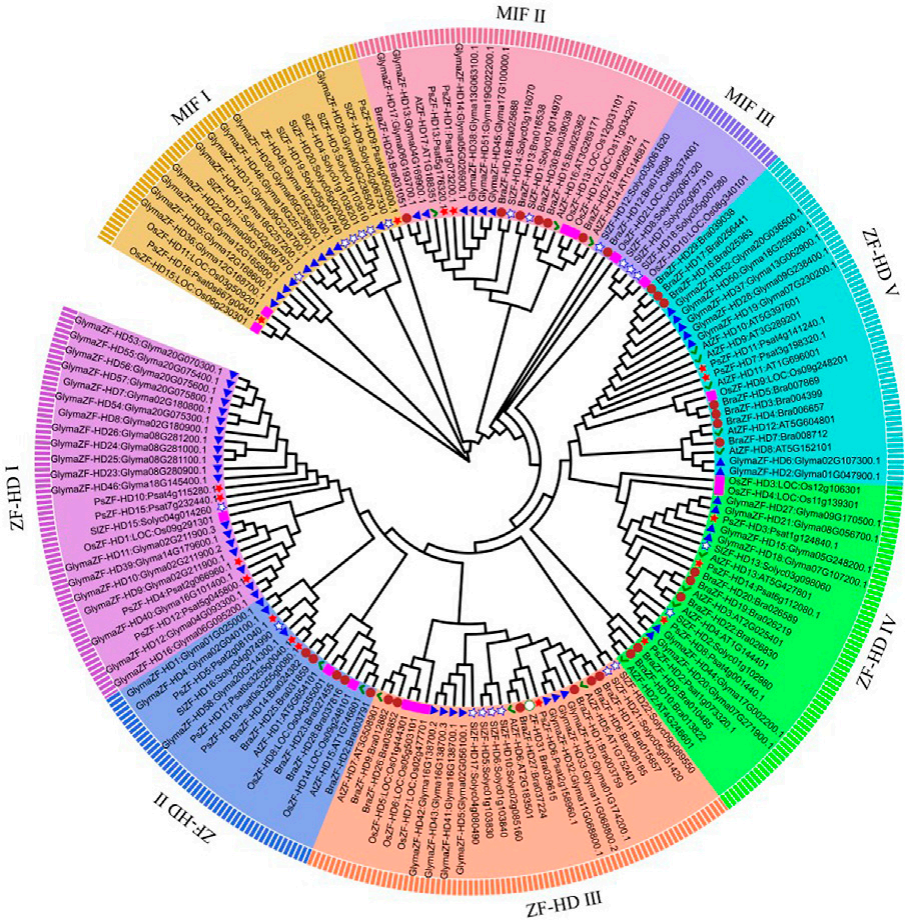
The phylogenetic tree of ZF-HD proteins from pea (*Pisum sativum,*Ps), Arabidopsis (*Arabidopsis thaliana,*At), Tamato (*Solanum lycopersicum,*Sl), Soybean (*Glycine max,,Glyma*), Chinese cabbage (*Brassica rapa,Bra*) and rice (*Oryza sativa,Os*). The phylogenetic Tree members showed as blot and accompany a pentagram.

The phylogenetic tree classified the ZF-HD proteins into two subgroups, MIF and ZF-HD. MIF was further categorized into three branches (MIFⅠ, Ⅱ, and Ⅲ), and the ZF-HD subgroup was further classified into five branches (ZF-HD I, II, IV, and V) (Figure 1). PsMIF2 and PsMIF4 were classified into the MIF I branch, and PsMIF1 and PsMIF3 were classified into the MIF II branch. MIF III does not contain pea ZF-HD family members; the ZHD I subpopulation contains PsZHD8, 12, 3, and 10; the ZHD II subpopulation contains PsZHD4, 13 and 14; the ZHD III subpopulation contains PsZHD5; the ZHD IV subpopulation contains PsZHD1, 7, 11 and two; and ZHD V subpopulation contains PsZHD6 and 9. This evolutionary tree differs individually from that formed by the pea PsZF-HD family protein sequences and domain motifs, but it is highly consistent with the evolutionary tree generated by the CDS. Notably, the ZF-HD family has not diverged in the evolution of monocot and dicot plants.

### 3.3 Chromosomal localization, gene duplication and ka/ks analysis

**FIGURE 2:**
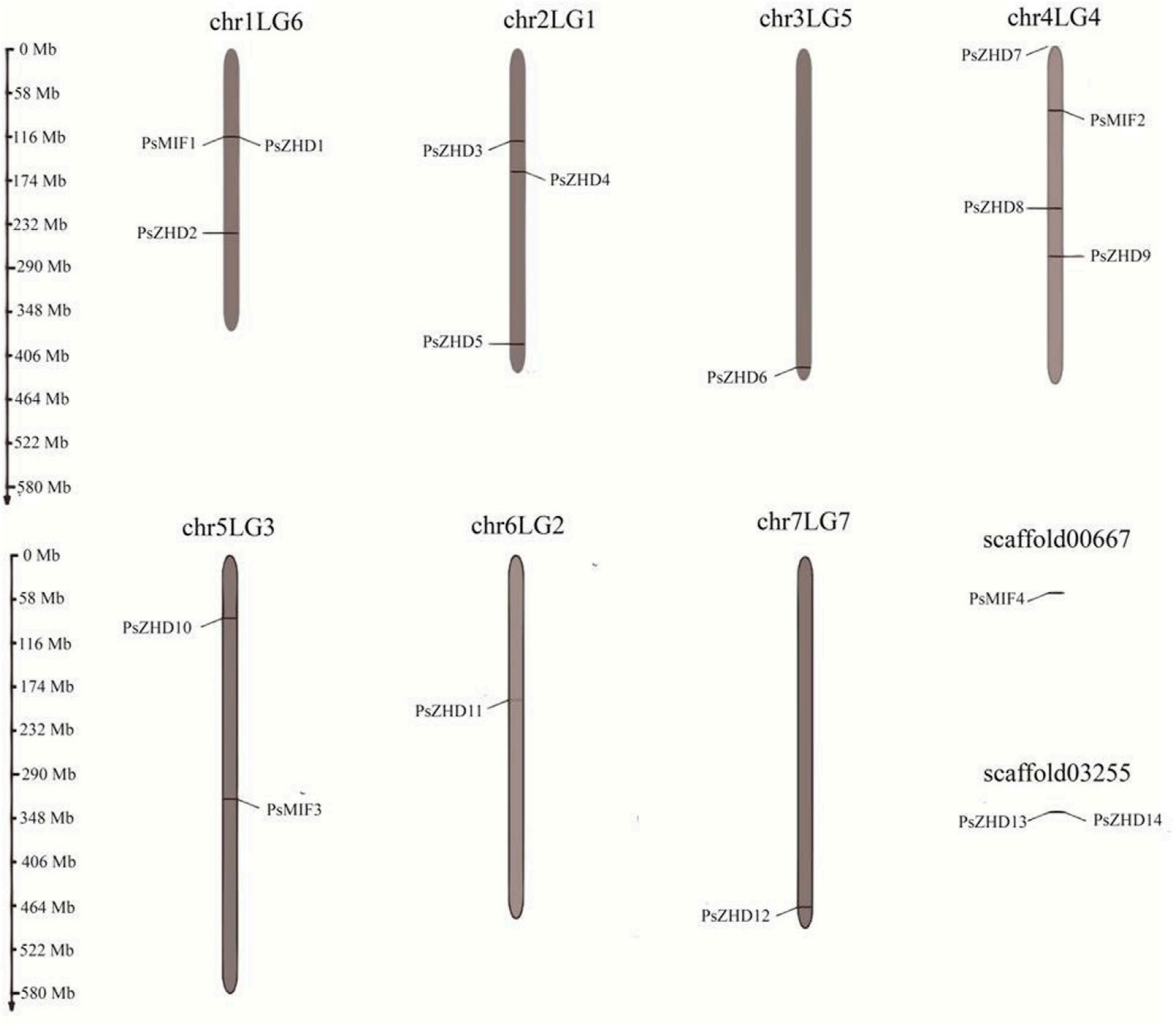
Physical distribution of *PsZF-HD* genes among seven chromosomes and scaffold.

The 18 *ZF-HD* family members in peas were unevenly distributed on seven chromosomes, and two large segments were not mounted on chromosomes, with different densities of gene distribution (Figure 2). Chromosome 1LG6 contained three *PsZF-HD* genes; 2LG1 contained three genes; 3LG5 contained one gene; 4LG4 contained the largest number of PsZF-HD members (four genes); 5LG3 contained two genes; 6LG2 contained one gene; 7LG7 contained one gene; sequence large segment Scaffold00667 contained one gene; and sequence large sequence segment Scaffold03255 contains one gene. PsZF-HD gene did not have large segment gene duplication events in seven chromosomes and two Scaffolds of pea, and only one pair of genes (Psat0s3255g0040.1 and Psat0s3255g0080.1) showed tandem duplication pairs. The ka and ks values were .206,821 and .1879, respectively, and ka/ks was 1.1007, indicating that the gene was under positive selection pressure during evolution.

### 3.4 Conserved motifs, conserved structural domains, and gene structure analysis

**FIGURE 3:**
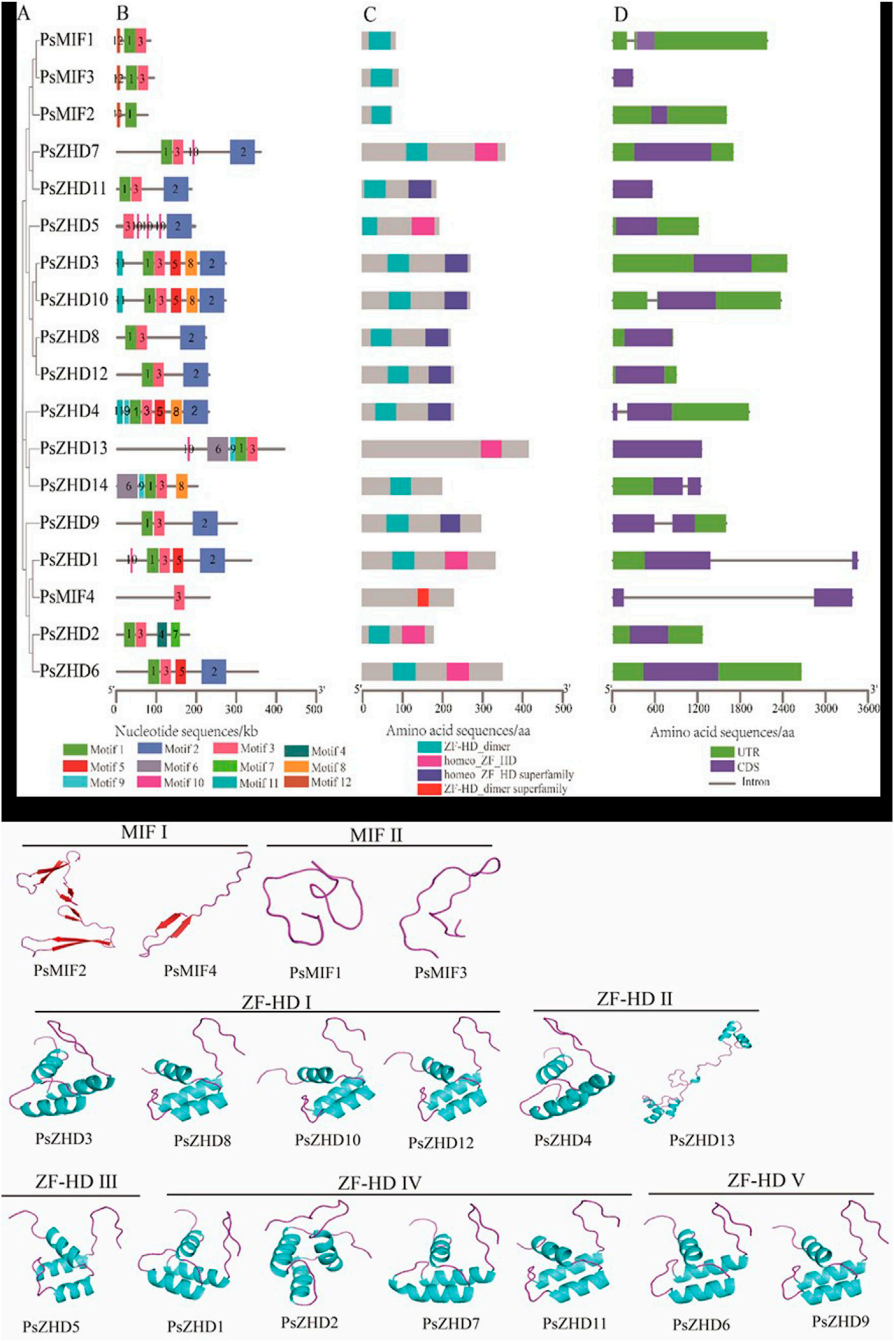
Phylogenetic relationship, conserved motifs, conserved domain and gene structure of the *PsZF-HD* genes. **(A)** An unrooted phylogenetic tree was constructed by the MEGA X based on Pea ZF-HD protein sequences using the Maximum likelihood method. **(B)** Conserved motif composition of the PsZF-HD proteins, and the colored box at the bottom represented the relative position of each conserved motif, Details are shown in Supplementary File S2. **(C)** conserved domain composition PsZF-HD proteins. **(D)** The CDS–UTR-introns structures of PsZF-HD genes were displayed by TBtools software.

Twelve Motifs were identified from the 18 PsZF-HD family members, namely motif1-12 (Supplementary File S3). Motif1 was present in all the PsZF-HD family members, except PsMIF4 and PsZHD5, and Motif3 was present in all the PsZF-HD family members, except PsMIF2. Motif2 was found in 11 members of the PsZF-HD family. The MIF family members had the lowest number of motifs; Motifs were classified as MIFⅠ. Motif12 is unique to the MIF members, and the ZHD family has more motifs. Motif9 is specific to ZHDII subgroup PsZHD13, 14, and 4 (Figures 3A). The conserved domain analysis (Figures 3B) showed that the 18 PsZF-HD family members contained four conserved domains, while the MIF subgroup members contained only one conserved domain, and all the ZHD subgroup members contained two different conserved domains. PsMIF4, which is classified in the MIF subgroup, contains a specific ZF-HD dimer superfamily domain, while the other 17 members contain an N-terminal ZF-HD dimer domain (rich in cysteine and histidine). PsZHD3, 8, 12, 4, and nine contain another hemeo ZF-HD superfamily domain. Structural analysis of PsZF-HDs genes showed (Figures 3C) that most members had no introns (13/18), and five members (PsZHD4, PsZHD14, PsZHD9, PsZHD1, PsZHD5) contained only one intron. The psMIF4 contained the longest intron and the longest untranslated region sequence. The highest matching templates with the PDB library were selected to construct and visualize 3D models of PsZF-HDs family proteins (Figure 3). psat4g050800.1 (3qdy.pdb), Psat5g176320.1 (6eu0.1. I.pdb), Psat1g073200.1 (2fqh.pdb), Psat0s3255g0040.1 2 (lbc.1.A.pdb), Psat0s667g0040.1 (1fr5.1.A.pdb), and the rest of the proteins use the same template 1wh7. pdb. There were significant structural differences between the MIF subpopulation and the ZF-HD subpopulation, while there was a high degree of structural similarity within the subpopulation. Members of the MIF subpopulation had no α-helix. MIFⅠ consisted of β-fold and a ring, while MIFⅡ had neither helix nor fold and consisted of a ring. In contrast, the ZF-HD subfamily was *β*-folded and had a more complex structure than the MIF members. Notably, PsZHD13, the 3D model, was significantly different from other ZF-HD subgroup members.

### 3.5 Analysis of structural elements of the promoter region of *PsZF-HD*

**FIGURE 4:**
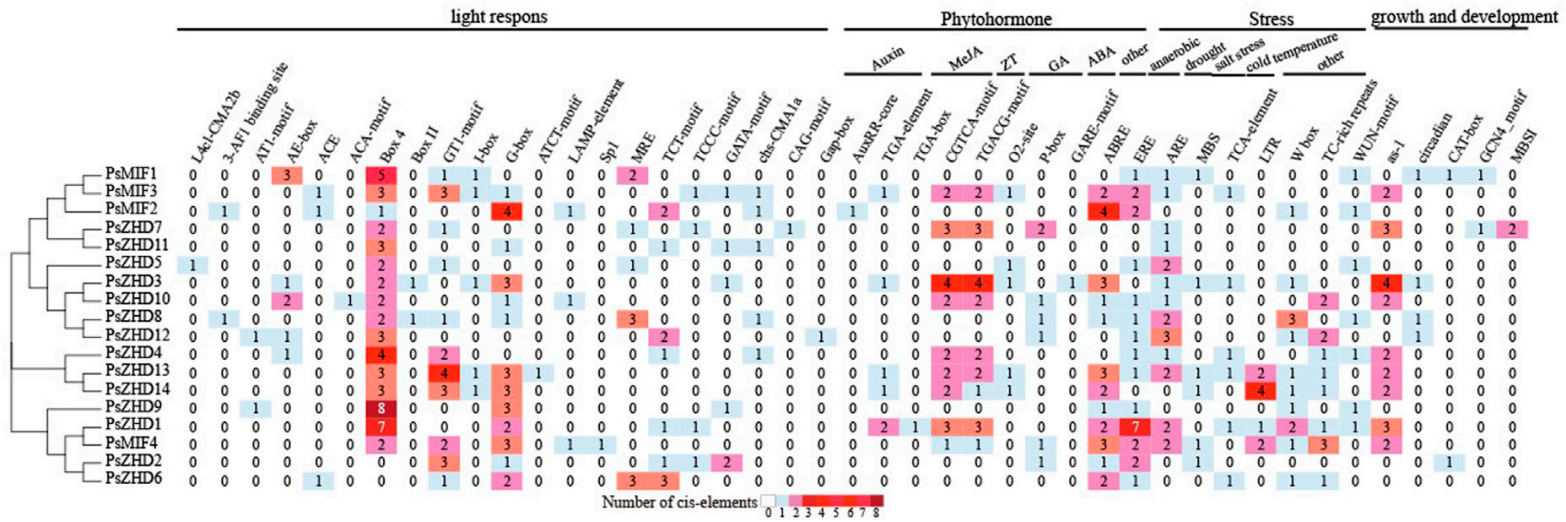
Analysis of Cis-acting elements of *ZF-HD* gene family in *P. sative*.

Cis-acting elements serve as molecular switches in the promoter regions of genes and important regulators of gene transcription during plant development in response to biotic/abiotic stresses and phytohormones. We extracted elements in the 1.5 kb promoter region upstream of the transcriptional start of PsZF-HD family members and filtered out unknown and untrustworthy elements to analyze 21, 10, 9, and 5 cis-acting elements involved in light, hormone, and stress responses and the regulation of growth and development, respectively (Figure 4). Of the 21 light response elements found in the 18 PsZF-HD family members, Box4, GT1-motif, and G-box had the highest coverage percentages at 16/18, 14/18, and 14/18, respectively. Fourteen members contained more than two light-responsive elements. The hormone-regulated response elements involved growth hormone, gibberellin, methyl jasmonate, zeatin, and abscisic acid, and the abscisic acid response elements were common in the *PsZF-HD* family, accounting for 14/18. The growth hormone AuxRR-core and TGA-box response elements were only present in *PsZF-HD*9 and 2, respectively. Gibberellin response element GARE motif existed only in *PsZF-HD*4. The other 16 *PsZ-HD* members contained more than two hormone response elements. PsZF-HD family genes contained stress response elements involving abiotic stresses, such as those induced by drought, low temperature, salt, and anaerobic factors. Of these, ARE anaerobic response elements had the highest distribution in the PsZF-HD family, reaching 13/18. PsZF-HD17 (except WUN motif element) and PsZF-HD2 (except MBS drought response element) contained six other stress response elements. *PsZF-HD*8 and 14 contained only anaerobic response elements. *PsZF-HD*3 contained only drought response element MBS. Five response elements regulate pea growth and development, none of which was found in PsZF-HD9, 6, 14, 7 and 11, while the other 13 PsZF-HD members contained at least one response element. The as-1 elements were distributed in the largest proportion of the *PsZF-HD* family at 1/2. Cluster analysis showed that some *PsZF-HD* members in the same branch had similar cis-elements. This suggests that the *PsZF-HD* family is involved in different developmental processes in response to abiotic stresses and hormonal regulation.

### 3.6 Inter-tissue differential expression analysis of PsZF-HDs genes

**FIGURE 5:**
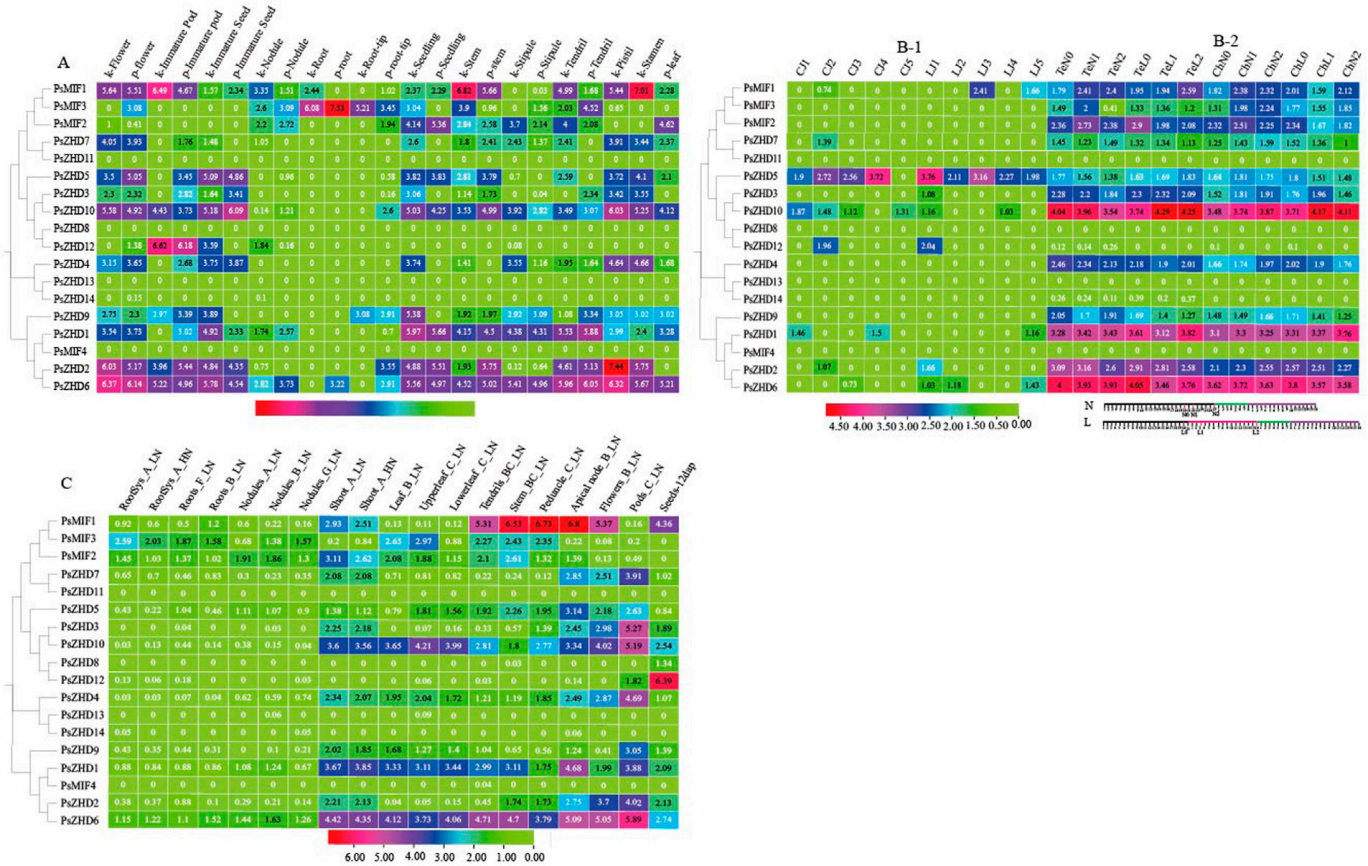
Expression patterns of *PsZF-HD*s in Different Tissues from Pisum sativum L. The heat map with clustering was created based on the FPKM value of *PsZF-HD*s. Differences in gene expression changes are shown in colour as the scale. **(A)** The expression pattern of *PsZF-HD* genes in the flower, immature pod, immature seed, nodule, root, root-tip, seeding, stem, stipule, tendril, pistil, stamen, leaf of Two pea varieties with significant phenotypic differences(Kaspa variety and Parafield variety). **(B-1)** Expression of *PsZF-HD*s gene in five stages of immature pod development. **(B-2)** The expression pattern of *PsZF-HD* genes in the leaf under different low-temperature stress. Scheme of the experiments and samplings of RNA-sequencing (RNA-seq) experiments:low temperature (L) treatment and control (N),low temperature in fuchsia, freezing in deepskyblue, and recovery period in darkviolet. **(C)** The expression pattern of *PsZF-HD* genes in different tissues of pea: The sampling points are shown in Supplementary File S3. The expression pattern of *PsZF-HD* genes in different tissues of pea: The sampling points are shown in Supplementary File S5.

To investigate the biological roles of *PsZF-HD*s, the expression levels of 18 *PsZF-HD*s genes were systematically examined in pea flowers, immature pods, immature seeds, roots, root tips, seedlings, stems, leaves, stipules, tendrils, stamens, post-flowering pods at five developmental stages and under the stress conditions of N deficiency and low temperature (Figure 5, Supplementary File S4). The aforementioned tissue contained *PsZHD*11, 8, 13, 14 and *MIF4* (Figure 5A). The other *PsZF-HD* members were differentially expressed in various tissues and demonstrated tissue specificity. However, they were similarly expressed in the same tissues of the two groups of phenotypically significantly different pea varieties (Kaspa and Parafield, WDZY-14 and WDZY-04) (Figure 5B-1). *PsZHD*6, 2, and 10 and *MIF*1 (except immature seeds) were significantly expressed in the aboveground reproductive organs. *PsZHD*6, 1, and 10 were significantly expressed in the aboveground nutritional organs, and *PsMIF*3 was highly expressed in the subterranean organs.

In the petals, the expression of all *PsZF-HD* members was upregulated, except for the aforementioned non-expressed genes, of which *PsZHD*6, 2, and 10 and *MIF*1 genes were highly expressed in that order, presumably relating to pea flowering regulation. The genes *PsZHD*12, 6, *MIF*1 and 10 were the most highly expressed in Kaspa and Parafield pea varieties during pod development. Moreover, *PsZHD*5 was consistently more expressed than the other members of *PsZF-HD*s at the five pod developmental stages of WDZY-14 and WDZY-04 pea varieties. *PsZHD*10, 6, five and two were sequentially highly expressed in immature seeds. *PsZHD6*, *MIF*1, and *MIF*3 were relatively highly expressed in the root nodules. *PsMIF*3 was specifically highly expressed in the roots and root tips, and its expression in roots was the second highest value of this family of genes in all the organs of peas. In the seedlings, *PsZHD*1, 6, 10, and two were sequentially highly expressed. The stems showed a sequential upregulation of *PsMIF*1, 6, 1, and 10 over the course of their development. The stipules showed exceptionally high expression of *PsZHD*6 and 1. The tendrils showed sequentially high expression of *PsZHD*6, 1, and 2. The 12 *PsZF-HD* members were highly expressed in the pistil and stamen, except *PsMIF*2, with *PsZF-HD*2 in the pistil showing the highest expression in all the organs of pea and *PsMIF*1 showing the third highest expression in the stamen. The expression of *PsZHD*6 in the leaves was relatively high. The expression of *PsMIF*3 in pea underground tissues was higher than other members of *PsZF-HD*s under a high-low N environment (Figure 5C), but it did not show a certain pattern during its development. *PsZHD*6, 1, 10 and *MIF*1 (except leaves) showed significantly high expression in aboveground nutritional tissues, with the highest expression of *PsMIF*1 in stems and terminal nodes in the low-N environment and the highest expression of *PsMIF*1 in pedicels in aboveground reproductive organs. The expression of the 17 *PsZF-HD*s members in the shoot organs was slightly higher in the low-N environment compared with the high-N environment.

This study analyzed the leaf transcriptome of two pea varieties (Te: freeze-sensitive spring dry bean variety, Ch: freeze-resistant winter forage variety) under control (20 °C day/14 °C night), low temperature (8 °C day/2 °C night) for 3 days, and low temperature (8°C day/2 °C night) for 16 days to clarify the mechanism of *PsZF-HD*s affecting low-temperature stress (Figure 5B-2). *PsZHD*11, 8, 13 and one were not detected in all experimental groups. Meanwhile, the expression values of *PsZHD*12 and 14 were close to or equal to 0, indicating that they were almost independent of leaf development. On the contrary, *PsZHD10,6,1* and one were highly expressed in the control and different low temperature environments, and their gene expression levels were ranked from high to low. Compared with the control, the gene expression of *PsZHD10* and *PsZHD10* was up-regulated. In contrast, *PsZHD*6, 2, *MIF*2 and *MIF*1 genes were down-regulated (except the *PsZHD*2 gene in ChN1 and ChL1 low-temperature treatment groups and the *PsMIF*1 gene in TeN2 and TeL2 low-temperature treatment groups), and their up- or down-regulation was less affected by the duration of low temperature. Other members of PsZF HDs did not show regular changes under the control and different low temperature durations.

### 3.7 Functional enrichment analysis of *PsZF-HD* gene and protein interaction network

GO enrichment analysis showed that the functions of ZF-HD family genes were mainly enriched in three levels: biological process (BP), cellular component (CC) and molecular function (MF). At the level of biological process, the target gene ZF-HD was significantly enriched in several GO items, such as the growth and development process (GO: 0032502), bioregulatory process (GO:0065007), metabolic process (GO: 008152), cell replication and reproduction, 11 genes were involved in biological metabolism, seven genes exercised biological management function, one gene had the function to resist At the cellular component level, most of the *ZF-HD* genes are the main components of the constituent cells, and a small number of *ZF-HD* genes are involved in the constitution of organs; and the molecular functions of the *ZF-HD* gene family are mainly enriched in the DNA binding function, which is one of the typical features of transcription factors. The results of GO enrichment showed that 18 PsZF*-HD* genes could be involved in rapid growth and development in peas.

## 4 Discussion

### 4.1 Evolutionary and structural characteristics of *PsZF-HD* genes in pea

The plant ZF-HD family genes play an important role in regulating plant growth and development and resisting stresses in a stressful environment. According to the bioinformatics analysis, the pea genome carried a total of 18 ZF-HD genes, with four *PsMIF* genes and 14 *PsZHD* genes similar to the categories of model plant Rice and Arabidopsis ZF-HD genes, respectively. However, the number of each subgroup in pea was different from that in rice and Arabidopsis. Previously, 58, 28, and 18 ZF-HD members were identified in soybean, wild soybean, and Tribulus alfalfa of the legume subfamily Pteridophyllaceae, respectively. In Chinese cabbage (Wang et al., 2016), tea (Zhou et al., 2021), buckwheat (Liu et al., 2019), maize (Jing et al., 2022), tobacco (Sun et al., 2021), and tomato (Hu et al., 2018), a total of 31, 18, 20, 24, 32, and 22 ZF-HD family members, respectively, have been reported. A total of 17 and 15 genes were identified in Arabidopsis and rice model crops, respectively. Although the number of the ZF-HD family members of the aforementioned plants are not less than that of pea, their genomes are much smaller (soybean, 1.15 Mb; alfalfa, 360 Mb; cabbage, 283.8 Mb; tea, 3.14 Gb; buckwheat, 489.3 Mb; tomato, 900 Mb. Previous studies have shown that ZF-HDs underwent a genetic expansion to differentiate between higher and lower plants. Most species undergo more than one genome contraction/expansion event during evolution, causing changes in gene numbers. A similar event has now been shown to occur in pea (Wang et al., 2016; Kreplak et al., 2019). Therefore, it is hypothesized that the gene duplication/loss events occurring in peas may be a key factor in the sparse distribution of *PsZF-HD*s genes in the genome.

The results of the biochemical analysis and phylogenetic tree showed that the ZF-HDs proteins were classified into two distinct subgroups (MIF and ZHD) and subdivided into three branches for MIF and five branches for ZHD (ZHD1-ZHDV) (Figure 1). This result is consistent with the previous reports on other crops (Liu et al., 2019; Zhou et al., 2021; He et al., 2022). The pea genome carried four PsZF-HD members classified into the MIF I subgroup and 14 members classified into the ZHD subgroup. Conserved motifs, protein structural domains, and 3D structures significantly differed between the MIF and ZHD subgroups. Members of the MIF subgroup contained the lowest number of motifs, which was unique to motif12. Meanwhile, the MIF subgroup contained only one conserved structural domain. Similarly, the ZHD subgroup contained only one conserved domain, and the ZHD subgroup had no motif12. However, both subgroups contained two different conserved domains. This is consistent with the previous proposal that the MIF subgroup contains only the ZF structural domain and lacks the C-terminal homology domain. In addition, the three-dimensional structures of the MIF and ZHD subgroups and the proteins within the MIF subgroup were significantly different. However, the members of the ZHD subgroup did not show significant structural differences among the branches. These results corroborate and support the correct classification of the PsZF-HD family, which has high evolutionary conservation. The present study did not show the evolutionary divergence of the ZF-HD family among monocots, which may be attributed to the small number of plants selected. Gene structure analysis may provide clues to gene family evolution (Lai et al., 2021). Most members of the pea PsZF-HD family are intronless, which matches the typical features of the ZF-HD family gene structure. It has been proposed that the loss of selective splicing of intronless genes facilitates the maintenance of the highly conserved sequence and functional stability of ZF-HD proteins during the evolutionary process. Moreover, it enables rapid transcription and translation in response to abiotic stresses (Shalmani et al., 2019). Furthermore, the five members of the PsZF-HD family contained only one intron, and we hypothesized that intron acquisition events occurred during the evolution of the pea *ZF-HD* gene. This provides further evidence that *ZF-HD* genes are subject to strong purifying selection, and it also suggests that members of the PsZF-HD family may have relatively conserved biological functions. However, the distinction between phase functions has not been made to date (Abdullah et al., 2018).

### 4.2 Analysis of cis acting elements of *PsZF-HD* gene promoter and its expression in different tissues at different growth stages and under abiotic stress

TFs are involved in the regulation of stress signals and expression of stress-responsive genes through various mechanisms, which depend on the presence of cis-acting elements in the promoter region. A growing body of evidence indicates that ZF-HD TFs play crucial roles in regulating various biological processes in plants (Hu et al., 2008; Khatun et al., 2017; Zhou et al., 2021). In our study, 45 cis-acting elements of the *PsZF-HD* with known biological functions were predicted and screened; these elements belonged to four categories: light response, stress, hormone, and growth and development, suggesting that the *PsZF-HDs* are also involved in different processes, such as photomorphogenesis, organ development, stress response, and hormone regulation, in pea plants. *ZF-HD* genes are differentially expressed in different tissues of different species and play an important role in plant growth and development (Khatun et al., 2017). Previous studies on *ZF-HD* genes have mostly focused on abiotic stresses and less on organ development. Previously, scholars analyzed the expression of ZF-HDs family genes in cabbage, buckwheat, and cucumber in their respective different organs and found significant spatiotemporal expression differences and tissue preference in different tissues (Liu et al., 2019). For example, buckwheat *FtZHD*10 and three were expressed only in the roots and *FtMIF*3 only in the flowers; the fruits showed high expression of *FtZHD*11/6/15/13 and no expression of *FtZHD*2. *FtZHD*1/2/4/5/7/9/12/16/17 and *FtMIF*2/3 were expressed more in the flowers than that in the other tissues; *FtZHD*1/6/11/12/15 were expressed more in the reproductive organs than that in the nutritional organs (Liu et al., 2019). Cucumber *CsMIF*1, *CsMIF*3, and *CsZHD*1 were highly expressed in the roots, flowers, and tendrils, respectively, and several *CsZF-HD* genes were significantly downregulated at the late stage of fruit development (Lai et al., 2021). We found that *PsZF-HD*s had a similar pattern as previously described. *PsZHD*11, 8, 13, and 14 and *MIF*4 were not expressed in the different tissues of the pea multiset transcriptome or were lowly expressed in the individual tissues. The other 13 *PsZF-HD*s genes were differentially expressed in the different tissues of pea. For example, in aboveground reproductive organs of pea, *PsZHD*6, 2, and 10 and *MIF*1 (except immature seeds) were significantly expressed; in aboveground nutritional organs, *PsZHD*6, 1, 10 were significantly expressed; and in the underground root system, *PsMIF*3 was highly expressed; in aboveground nutritional organs, *PsZHD*6, 1, 10 were significantly highly expressed; and in the underground root system, *PsMIF*3 was specifically highly expressed. It has been proposed that *Arabidopsis thaliana AtZHD*5 is highly expressed in leaves with the same branches as tobacco *NtZF-HD*22 and *NtZF-HD*2 and *Arabidopsis thaliana AtZF-HD*8 is highly expressed in flowers and leaves(Sun et al., 2021). In this study, high expression of *PsZHD*6 in leaves of the same branch as *AtZF-HD*8 was found. It indicates that *PsZHD*6 is involved in regulating the growth and development of pea leaves. Seedlings highly expressed genes (*PsZHD*1, 6, 10), stems (*PsMIF*1, 6, 1, 10), stipules (*PsZHD*6 and 1), tendrils (*PsZHD*6, one and 2), and leaves (*PsZHD*6 and *MIF*2). Notably, the pod transcriptome data published by Sudheesh et al. (2015); Yang et al. (2019) differed significantly, with the former showing high expression of *MIF*1/10/12/2/6 genes in all immature pods, while the latter showed only *PsZHD*5-specific high expression at five periods of pod development. In the floral organs (petals, pistils, and stamens), most *PsZF-HD* members were expressed at high values, with *PsZF-HD*2, *PsMIF*1, 6 and 10 being significantly overexpressed in the floral organs in that order, presumably in relation to pea flowering regulation. Previous studies have shown that most of the *Arabidopsis thaliana* ZF-HD family genes are expressed in the floral organs and overlap in regulating flower development (Shalmani et al., 2019). *BraZF-HD*30 is specifically expressed in the flower tissue of Chinese cabbage (Wang et al., 2015).

*ZF-HD* genes play key roles in response to biotic and abiotic stresses. For example, *Arabidopsis thaliana AtZF-HD*4 is up-regulated in response to drought, cold stress, and salinity. Most *BraZF-HD* genes in cabbage are induced by photoperiod, vernalization, low temperature and abiotic stresses (Wang et al., 2016). *ZF-HD*3 was gradually up-regulated, and *ZF-HD*15 was down-regulated by cold stress 0–24 h in a tea tree(Zhou et al., 2021). Tomato *SlZHD*13 was up-regulated under drought, and salt stress (Khatun et al., 2017), and silencing the *SL-ZH*13 gene reduced its resistance to cold and salt stresses (Zhao et al., 2018; Zhao et al., 2019). In this study, we analyzed the transcriptome results of pea leaves under low-temperature stress at 3 days and 16 days. We found that low temperature induced up-regulation of *PsZHD*10 and one genes and down-regulation of *PsZHD*6, 2, *MIF*2 and *MIF*1 genes, but their up- and down-regulation was not significant. Under low N stress, *PsZHD*6, 1, 10 and *MIF*1 (except leaves) were significantly highly expressed in aboveground nutrient tissues, with *PsMIF*1 showing the highest expression in stems, terminal nodes and pedicels. The expression of 17 *PsZF-HD*s members was slightly up-regulated in shoots. Similar results have been verified in other crops (Wang et al., 2016; Khatun et al., 2017). The PsZF-HD members in the same branch have more similar types of action elements. For example, the ZHD V branch members PsZF-HD7 and 11 are highly similar in hormone, stress and growth and development-related action elements, and the three members of the ZHD IV branch (except PsZF-HD2) are highly similar in their response elements. The ZHD III branch contains only PsZF-HD6, which contains the least number of active elements and is divided into a single branch. 16 genes were highly similar in some light-responsive and hormone-responsive element types. Overall, their expression was detected during pea organ development and morphogenesis; these genes may not be associated with pea organ development or light-induced morphogenesis. Overall, Some *PsZF-HD* family genes in pea are involved in regulating the response to stress, focusing on the functional verification of the above genes will help uncover the stress resistance of peas and play an important role in promoting the improvement of crop stress resistance.

## Conclusion

18 *PsZF-HD* genes, including 14 ZHD genes and four MIF genes, were identified in the entire pea genome, and their Conserved motif, Conserved domain, structures and expression pattern of in various tissues, different stages of pod development, together with the expression patterns of BraZF-HD genes under low temperature and nitrogen stress were analyzed, provided a basic resource for the examination of the molecular regulation of pea development and stress resistance. Our findings is the first systematic and comprehensive analysis of *ZF-HD* genes in pea, it provides clues for revealing the potential roles of *PsZF-HD* genes in Morphogenesis and tissue development and stress tolerance of Pea.

## Data Availability

The original contributions presented in the study are included in the article/Supplementary Material, further inquiries can be directed to the corresponding authors.
