# Supplementary material for: Genome-wide identification and expression analysis of the ZF-HD gene family in pea (Pisum sativum L.)
Source: Front Genet. 2023 Jan 5;13:1089375. doi: 10.3389/fgene.2022.1089375 (PMC9849798; doi:10.3389/fgene.2022.1089375)
Supplement: Supplementary file 1 [file Table1.DOCX]

>PsMIF1 Psat1g073200.1 domain:19-70

VKYGECQKNHAANVGGYAVDGCREFMANGEEGTDGALTCAACGCHRNFHKRE

>PsZHD1 Psat1g073320.1 domain:76-129

VTYKECLKNHVATLGGHALDGCCEFMPSPTATSDDPASIKCAACGCHRNFHRRE

>PsZHD2 Psat1g124840.1 domain:19-70

IYRECLRNHAASLGSYATDGCGEFTVDDTSVSAANSLQCAACGCHRNFHRKI

>PsZHD3 Psat2g066960.1 domain:65-117

VRYRECQKNHAVSFGGHAVDGCCEFISAGEEGTLEAVICAACNCHRNFHRKEI

>PsZHD4 Psat2g081040.1 domain:34-84

RYRECLKNHAVGIGGHALDGCGEFMPAGNEGSLESLKCAACNCHRNFHRKE

>PsZHD5 Psat2g158960.1 domain:1-39

MGSHVVDGCGEFMPSGEEGTPQSFICAACDCHRNFHRKH

>PsZHD6 Psat3g198320.1 domain:79-132

VTYKECLKNHAANLGGHALDGCGEFMTSPTATSADPTSLKCAACGCHRNFHRRE

>PsZHD7 Psat4g001440.1 domain:111-163

VKYRECLKNHAAAMGGNATDGCGEFMPSGEQGSIEALICSACHCHRNFHRKEI

>PsMIF2 Psat4g050800.1 domain:22-70

VRYGECQKNHAATSGGYAVDGCMEFMASSGDDALICAACGCHRNFHRRK

>PsZHD8 Psat4g115280.1 domain:22-72

EYKECRKNHAFKIGCYAIDGCCEFLPAGTEGTVEFFKCAACGCHRNFHRKE

>PsZHD9 Psat4g141240.1 domain:62-115

VSYKECLKNHAASLGGHALDGCGEFMPSSSVNPNDPRSLKCAACGCHRNFHRRE

>PsZHD10 Psat5g045800.1 domain:69-121

VRYRECQKNHAVSIGGHAVDGCCEFLAAGEEGTLEAVICAACNCHRNFHRKEI

>PsMIF3 Psat5g176320.1 domain:23-76

IRYGECQKNHAANIGGYAVDGCREFMASTGEEGTGGALTCAACGCHRNFHRREV

>PsZHD11 Psat6g112080.1 domain:7-59

VKYKECLKNHAAAIGGNATDGCGEFMASGDNDTLEALNCCACDCHRNFHRKEI

>PsZHD12 Psat7g232440.1 domain:64-114

MFKECRRNHASGIGGFAVDGCCEFLPAGIEGTIEFFKCAACNCHRNFHHKE

>PsMIF4 Psat0s667g0040.1 domain:141-165

TATSADPTSLKCAACGCHHNFHRRE

>PsZHD13 Psat0s3255g0040.1 domain:298-348

RYRECLKNHVVEIDGHALDGCGEFMPAGNEGSLESLKCVRCNCHCNFHRKE

>PsZHD14 Psat0s3255g0080.1 domain:71-121

RYRECLKNHVVGIGGHALDGCGEFMPAGNEGSLELLKRVACNCHRNFHCKE
