# Supplementary material for: Genome-wide identification and expression analysis of the ZF-HD gene family in pea (Pisum sativum L.)
Source: Front Genet. 2023 Jan 5;13:1089375. doi: 10.3389/fgene.2022.1089375 (PMC9849798; doi:10.3389/fgene.2022.1089375)

Motif1: E-value= 6.7e-2831, Sites= 88, Width(aa)= 49, Log Likelihood Ratio=8395
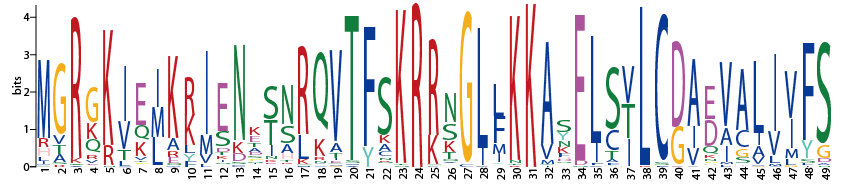


Motif2: E-value= 7.9e-1024, Sites= 16, Width(aa)=  100, Log Likelihood Ratio= 3795


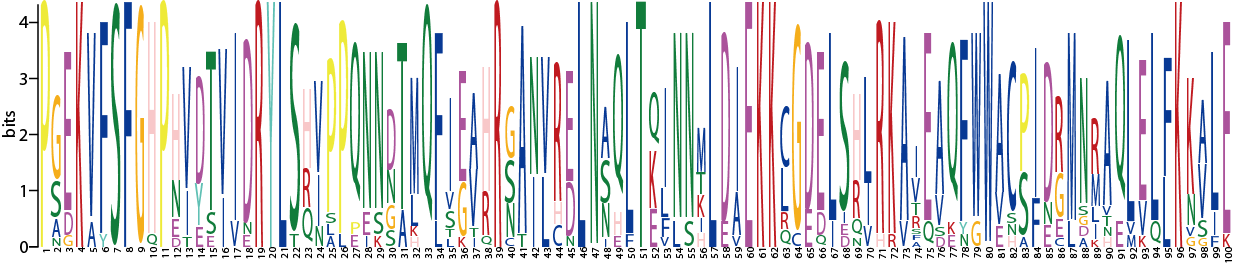


Motif3: E-value=2.3e-1020, Sites=43, Width(aa)=71, Log Likelihood Ratio=4169
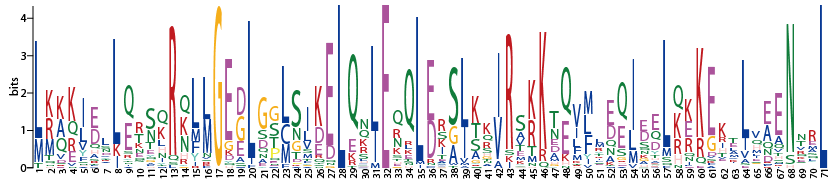


Motif4: E-value=4.4e-317, Sites=12, Width(aa)=78, Log Likelihood Ratio=1797
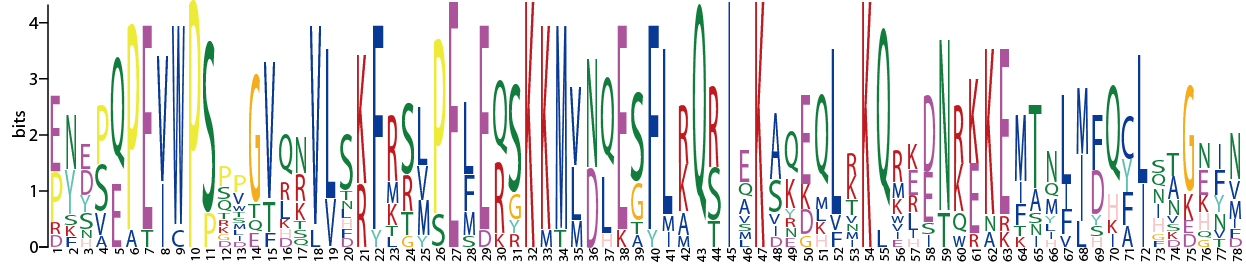


Motif5: E-value= 6.2E-202, Sites=48, Width(aa)=15, Log Likelihood Ratio=1128
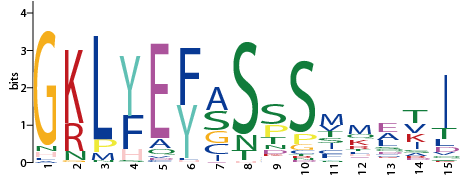


Motif6: E-value= 5.3E-160, Sites= 11, Width(aa)=29, Log Likelihood Ratio=780


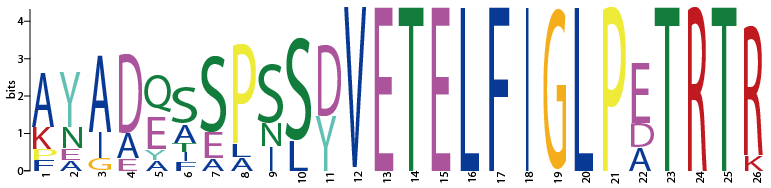


Motif7: E-value= 1.2E-101, Sites= 9, Width(aa)=26, Log Likelihood Ratio=573


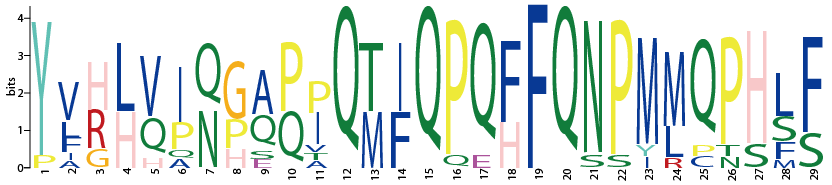


Motif8: E-value= 9E-81, Sites=4, Width(aa)=79, Log Likelihood Ratio=784


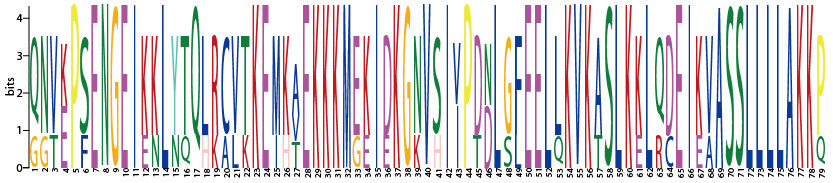


Motif9: E-value= 2.7E-73, Sites=6, Width(aa)=29, Log Likelihood Ratio=458


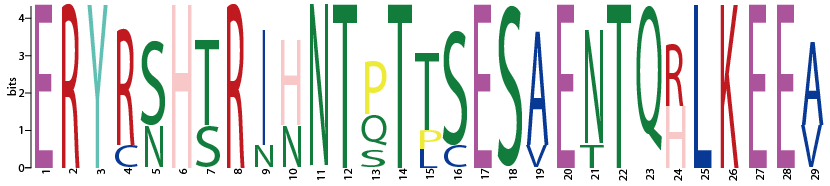


Motif10: E-value= 1.9e-064 , Sites=28, Width(aa)=15, Log Likelihood Ratio=623


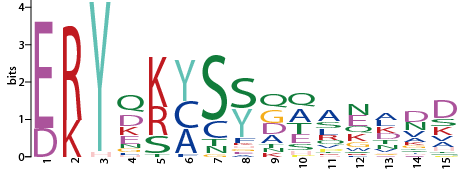


Motif11: E-value= 2.1E-58 , Sites=17, Width(aa)=29, Log Likelihood Ratio=703


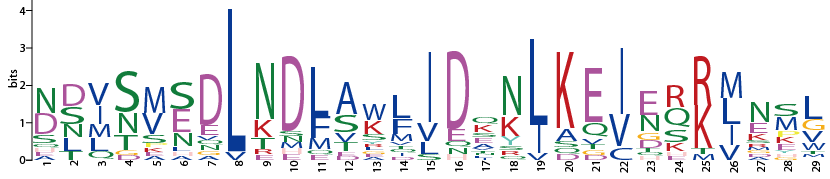


Motif12: E-value= 3.5E-50 , Sites=6, Width(aa)=29, Log Likelihood Ratio=416


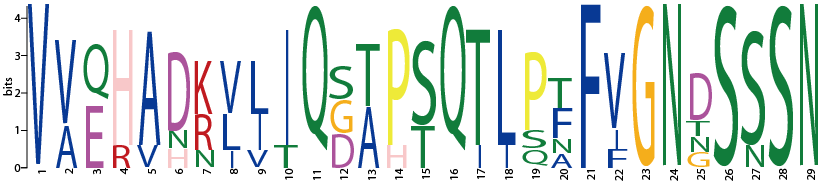

Supplement: Supplementary file 2 [file Table3.DOC]
