# Supplementary material for: Genome-wide identification and expression analysis of the ZF-HD gene family in pea (Pisum sativum L.)
Source: Front Genet. 2023 Jan 5;13:1089375. doi: 10.3389/fgene.2022.1089375 (PMC9849798; doi:10.3389/fgene.2022.1089375)
Supplement: Supplementary file 3 [file Table5.DOC]

| **Description** | **Organ** | **Stage** | **Nutrition** |
| --- | --- | --- | --- |
| RootSys_A_HN | Root system | A | High-nitrate,hydroponics |
| RootSys_A_LN | Root system | A | Low-nitrate,hydroponics |
| Root_B_LN | Roots | B | Low-nitrate,hydroponics |
| Root_F_LN | Roots | F | Low-nitrate, aeroponics |
| Nodule_A_LN | Nodules | A | Low-nitrate,hydroponics |
| Nodule_B_LN | Nodules | B | Low-nitrate,hydroponics |
| Nodule_G_LN | Nodules | G | Low-nitrate, aeroponics |
| Shoot_A_HN | Shoot | A | High-nitrate,hydroponics |
| Shoot_A_LN | Shoot | A | Low-nitrate,hydroponics |
| Leaf_B_LN | Leaves | B | Low-nitrate,hydroponics |
| LowerLeaf_C_LN | Lower leaves^a^ | C | Low-nitrate,hydroponics |
| UpperLeaf_C_LN | Upper leaves ^b^ | C | Low-nitrate,hydroponics |
| Tendril_BC_LN | Tendrils | B + C | Low-nitrate,hydroponics |
| Stem_BC_LN | Stems | B + C | Low-nitrate,hydroponics |
| Peduncle_C_LN | Peduncles | C | Low-nitrate,hydroponics |
| ApicNode_B_LN | Apical node | B | Low-nitrate,hydroponics |
| Flower_B_LN | Flowers | B | Low-nitrate,hydroponics |
| Pods_C_LN | Pods ^c^ | C | Low-nitrate,hydroponics |
| Seeds_12dap | Seeds | E | High-nitrate, pots |
| Seed_5dai | Seeds | D | Water |

TableS3 Expression of PsZF-HD gene in different tissues and organs of pea at different development stages under high and low nitrogen treatment

Stage A represents 7–8 nodes, 5–6 opened leaves; stage B represents the start of flowering; stage C represents 20 days after the start of

flowering; stage D represents germination, 5 days after imbibition; stage E represents 12 days after pollination; stage F represents 8 days

after sowing; stage G represents 18 days after sowing, i.e. 10 days after inoculation.

a Nodes below the flowering node (N-1, N-3, N-5).

b Nodes above the flowering node (N+1, N+3, N+5).

c Young, shiny, green 2 cm pods.
