# Supplementary material for: Genome-wide identification and expression analysis of the ZF-HD gene family in pea (Pisum sativum L.)
Source: Front Genet. 2023 Jan 5;13:1089375. doi: 10.3389/fgene.2022.1089375 (PMC9849798; doi:10.3389/fgene.2022.1089375)
Supplement: Supplementary file 4 [file Table2.DOCX]

>PsMIF1_Psat1g073200.1

MRKRQVVLRRSEEPQRSVVKYGECQKNHAANVGGYAVDGCREFMANGEEGTDGALTCAAC

GCHRNFHKRELVETESVSECSNGT*

>PsZHD1_Psat1g073320.1

MMEVLTTTTTATNTTPLTTISKPSEPDSENPTRIQQQQPVNTTTSNITKPLSFSNGVLKR

HHPNTHNHHNNNVVAVTYKECLKNHVATLGGHALDGCCEFMPSPTATSDDPASIKCAACG

CHRNFHRREPEEPISTVFEYQPHHRHHPPPPPLFQSRSPSSPSPPPISSYPSAPHMLLAL

SGAGVGLSIPPENTAAPLNLGSPMGASRKRFRTKFSQEQKDKMHEFAERLGWKMQKRDEE

MVNGFCNEVGVDRSVLKVWMHNNKNTLGRKLSDHGNGDGDAVRSAVDGVSDGGFGAHEND

NDGINVAASVFTKIAMSYDYFKNLNPTNFKHNLI*

>PsZHD2_Psat1g124840.1

MEGGGMLSSSSSPHSQNYIYRECLRNHAASLGSYATDGCGEFTVDDTSVSAANSLQCAAC

GCHRNFHRKITATYATMARDNAIVAVSDQVMEYSGGGDGRRKRYRSKFTADQKEKMLGFA

EKLGWKLQRKELDEEIERFCESVGVSRQVFKVWMHNHKNSCFSNSSDPSAGNANSSLTQ*

>PsZHD3_Psat2g066960.1

MEFDEHEEEHEEEMEIPETPMPVPASYDSLGNSASRSKISGGVDGRKGSGNGNGNSNSGG

FSSTVRYRECQKNHAVSFGGHAVDGCCEFISAGEEGTLEAVICAACNCHRNFHRKEIDGE

TVSSCQRQQPPPPPQPYHHHHNQFPPYYHRGPPSTSGYLHHHLATPVAHHRPLALPASAS

GGGFSREEDDISNPSSSGGGGNGSGGSKKRFRTKFTQEQKEKLLAFAEEHGWRIQKQDEA

AIEQFCAENCVKRNVLKVWMHNNKNTLGKKP*

>PsZHD4_Psat2g081040.1

MEFEDHEEQEEELCMGAASYDSNINIGVGNGKARYRECLKNHAVGIGGHALDGCGEFMPA

GNEGSLESLKCAACNCHRNFHRKESPDFTAGDPFLLTHHHHHALPQPQFAAYYRTPAGYL

HVSGQQRTGTLALPSTSGGGGGGGTQSTREDQDEDVSNPSGGGSSKKRHRTKFTVEQKDK

MLELAEKLGWRIQKHDEGLVQEFCNESGVKRHVLKVWMHNNKHTLGKKP*

>PsZHD5_Psat2g158960.1

MGSHVVDGCGEFMPSGEEGTPQSFICAACDCHRNFHRKHVQLPQQQQEQQHQQQTQHVVN

NGHVQFNTPSSSSQRFSHPTTSGHVPPLMMTFGSGPAESSSEDLNMLGAQFSIQTQQQQQ

QQVSKKRIRTKFSQLQKDKMMEFAERIGWKIQKHDEQEVQQFCSQVGIKRQVFKVWMHNN

KQAMRKTGNVSLA*

>PsZHD6_Psat3g198320.1

MDITPTQTTTTTPLPTILTTSTTTLTKSPEHETETPTKITTPTTTTTPKISSFSNGVLKR

LHNHHHHHLNNHHNNLVTVTYKECLKNHAANLGGHALDGCGEFMTSPTATSADPTSLKCA

ACGCHRNFHRREPEEPHLSTTTHVIEYQPHHRHQPLPPPPFSTRSPNSSSPPPISSTYYP

SAPHMLLALSTALPENVSAPNSAMISPGSHTRKRFRTKFTQDQKEKMLKFAEKVGWKMQK

KDEDYVHEFCNEICVDRSVLKVWMHNNKNTFAKKDNNINNLNDIDNNLNNINNEVNVGVK

SFLPLENEEHNINNINKGETKLEIHTHNHNHNHHYQNDVGVTVRANGSSSS*

>PsZHD7_Psat4g001440.1

MELSSTQDGEIQIPSNNYVATAHGVSANGHGHHHHHHMIHHHAPPPPPPLLHHHNNIITS

SSAAAVPVAAVVPQSQNPSINGTTTTTTNSTSVELDLDQSSYKKPLSQVAVKYRECLKNH

AAAMGGNATDGCGEFMPSGEQGSIEALICSACHCHRNFHRKEIEGETDEENYPHHHNSPF

NFNFNVNRQQQQHHSMRKFMLPLSDQPLGLGYHHHHTTPNSTSAAVTVTGNNNNNNNILP

SRAVAPPPHGHIIMPFNYNIPSESDEQEEHGRTQSHHQQVVMKKRFRTKFTQEQKEKMLN

FAEKVGWKFQKQEESLVQQFCAEIGVKRRVLKVWMHNNKHNLAKKQHIDISTDNLPTP*

>PsMIF2_Psat4g050800.1

MKKRQVVVVVKSNNSSSSRNVVRYGECQKNHAATSGGYAVDGCMEFMASSGDDALICAAC

GCHRNFHRRKVESAD*

>PsZHD8_Psat4g115280.1

MVIASNDDEENLMNVQSHTKTEYKECRKNHAFKIGCYAIDGCCEFLPAGTEGTVEFFKCA

ACGCHRNFHRKETIVVTVEQALSPLNINRFPQPTPISTVFQTSTGYVHVNGPPRGAIIYP

ALPSAVVHGREHLSREKIEDNVCVVESINGDGEGSSNSKKRFRTKFTHEQRKKMFDFAVK

SGWKIPKKDENLVEEFCNEIGVKCQILKVWMNNNKYTLGERH*

>PsZHD9_Psat4g141240.1

MDMTSRNIDVDSQTPPSIPQPNNNNNITPTTTLLKSSPFTNGTHKRHLPTTTTTPPPPSM

VVSYKECLKNHAASLGGHALDGCGEFMPSSSVNPNDPRSLKCAACGCHRNFHRREPQEQH

IVPTTNTTTVNATTNTTTNATTNPTFLNCIYTPASTTVPPPQPPPPLQLPHRGPISQSTS

PSQSTSPTSSPSPTTKFSKEQKEMMYGFSEKLGWRMQKGDDGMVQKFCNDVGVSRGVFKV

WMHNNKNLRKRPESEVGIATPISDNKNNNNNDGSNNVIHMDEDGCVNVHVSSVNVLSS*

>PsZHD10_Psat5g045800.1

MEFDEHEEDQEEEEEEEEEMGFPVTPVAGYDSLGNSGVRSKMSGGTMETDGAAVIPTGGV

LKNGPKGTVRYRECQKNHAVSIGGHAVDGCCEFLAAGEEGTLEAVICAACNCHRNFHRKE

IDGEVISHNHQPRSHSQTQYHHHQLSPYYHRAALPPPPGYHQLLTPPPVSHHRPLALPPV

ASSGGFSREDDDVSIPSSSGGGGSGTKKRFRTKFTQEQKDKMLAFAEGIGWRIQKEDEAA

IEQFCAENYIKRNVLKVWMHNNKHTLGKKP*

>PsMIF3_Psat5g176320.1

MKKRQVVLKRDVAKTTSSITRNIRYGECQKNHAANIGGYAVDGCREFMASTGEEGTGGAL

TCAACGCHRNFHRREVQTEVVCEYSPPNSAR*

>PsZHD11_Psat6g112080.1

MEKKLVVKYKECLKNHAAAIGGNATDGCGEFMASGDNDTLEALNCCACDCHRNFHRKEIV

YDSQNYALSLIPDHNINAPFLAHLSPIKSESNSPSDQSYYEKDCIKEVENRTEKMIKKRC

RTKFTKEQKEKMLCFAEKAEWKIQKLEESVVQMFCQEIGIKRRILKVWMHNNKNTFAKRN

VSSII*

>PsZHD12_Psat7g232440.1

MLFSIPLFVFFSSICFLTEQMKNESHDEEITTFQNNKQANNVETMEISNHQVDEPILNFQ

RKEMFKECRRNHASGIGGFAVDGCCEFLPAGIEGTIEFFKCAACNCHRNFHHKELVTGDL

TNFPANTVYYPSPTPISTIFPIHDYIHMTGSSRGTINGGEERLTSFKKRFRTKFSREQKE

KMFNFAVKLGWKVRKQDKNAVEKFCNKIGVKFQVFKVWILNNKHTIGKQT*

>PsMIF4_Psat0s667g0040.1

MRHELLVVVREGATGLGDGTCDCPKLGVGLEARCGAWSSMWGLELDVGLMGRYTTTPKHK

TTPFITPTFIILHPLAKTPSPPSSFSPATPNYPFVHLPTNIITVPSPEEHQHAVNPLLRP

YSDDNHHPICTYHNPPSISATATSADPTSLKCAACGCHHNFHRREPEEPPLSTATHTHAS

PRLRFQHIKAPTPPQIHFSNSDAVRPSPKIASRFLFRHRNRPIQSLHHFR*

>PsZHD13_Psat0s3255g0040.1

MSNTTQLSTHPSQSTKKPQQEQTQDTCCYSIQTTHFLITHSEARLKKLVDTIGEEVHTSS

IFVKPEDSSFAKLGDKMSDDSGLGRGGYDWNTEDELEIESFHSSCITVPYGQISGSRSLE

EISFAAGPSNTEVFDSLINMGFHPEMVAKVIQEYCEENEHKLVKEFLTYQELERFSQQQQ

QVEPDPISSEYAASSWDDSSDEDDSSNEEIPKSPSKNDDTLLSLVKMGFNEEEALMAVER

LGPNSSLERTPTTSVHCSSAVVTHSNNNNENSNSNNNNNNGNSNNSNINIGVGNGKARYR

ECLKNHVVEIDGHALDGCGEFMPAGNEGSLESLKCVRCNCHCNFHRKESPDFTVGDPFLL

NTPPPPCATSAAICGLLSNSGWVLACFRTAEDRDACSTVDLRRRRRWRDTEYEGGSR*

>PsZHD14_Psat0s3255g0080.1

MGFNEEEALMAVERLGPNSSLERTPTTSVHYSSTVVTHSNNNNGNSNSNNNNNGNSNNSN

INIGVGNGKARYRECLKNHVVGIGGHALDGCGEFMPAGNEGSLELLKRVACNCHRNFHCK

ESPDFTAGDPFLLNTPPPPCRTGTLTLLSTSGGGGGGGTQSTREDQDEDVLNPSGGGSSK

KRRRTKFTVEQKDSLFISMW*

>AtZF-HD4_AT1G14440.1

MEIASQEDHDMPIPLNTTFGGGGSHGHMIHHHDHHAANSAPPTHNNNNTTQPPPMPLHGN

GHGNNYDHHHHQDPHHVGYNAIIKKPMIKYKECLKNHAAAMGGNATDGCGEFMPSGEDGS

IEALTCSACNCHRNFHRKEVEGELAATAMSPYHQHPPHRKLMLNHQKIRSAMPHQMIMPI

GVSNYRYMHNNSESEDFMEEDGVTTASRSLPNLPYNQKKRFRTKFTPEQKEKMLSFAEKV

GWKIQRQEDCVVQRFCEEIGVKRRVLKVWMHNNKIHFSKKNNINLEDNDNEKINNLNNVD

LSGNNDMTKIVP

>AtZF-HD14_AT1G14687.1

MQSTCVYRECMRNHAAKLGSYAIDGCREYSQPSTGDLCVACGCHRSYHRRIDVISSPQIN

HTRFPFTSLRRVKQLARLKWKTAEERNEEEEDDTEETSTEEKMTVQRRRKSKFTAEQREA

MKDYAAKLGWTLKDKRALREEIRVFCEGIGVTRYHFKTWVNNNKKFYH

>AtZF-HD17_AT1G18835.1

MKKRQVVIKQRKSSYTMTSSSSNVRYVECQKNHAANIGGYAVDGCREFMASGGDDALTCA

ACGCHRNFHRREVDTEVVCEYSPPNANN

>AtZF-HD11_AT1G69600.1

MDLSSKPQQQLLNSLPIAGELTVTGEMGVCYKECLKNHAANLGGHALDGCGEFMPSPTAT

STDPSSLRCAACGCHRNFHRRDPSENLNFLTAPPISSPSGTESPPSRHVSSPVPCSYYTS

APPHHVILSLSSGFPGPSDQDPTVVRSENSSRGAMRKRTRTKFTPEQKIKMRAFAEKAGW

KINGCDEKSVREFCNEVGIERGVLKVWMHNNKYSLLNGKIREIEHGLCLNTHSNDGDGSS

SS

>AtZF-HD15_AT1G74660.1

MMKKRQMVIKQRSRNSNTSSSWTTTSSSSSSSEISNVRYVECQKNHAANIGGYAVDGCRE

FMAAGVEGTVDALRCAACGCHRNFHRKEVDTEVVCEYSPPNA

>AtZF-HD5_AT1G75240.1

PSSFSISAAAKPTVRYRECLKNHAASVGGSVHDGCGEFMPSGEEGTIEALRCAACDCHRN

FHRKEMDGVGSSDLISHHRHHHYHHNQYGGGGGRRPPPPNMMLNPLMLPPPPNYQPIHHH

KYGMSPPGGGGMVTPMSVAYGGGGGGAESSSEDLNLYGQSSGEGAGAAAGQMAFSMSSSK

KRFRTKFTTDQKERMMDFAEKLGWRMNKQDEEELKRFCGEIGVKRQVFKVWMHNNKNNAK

KPPTPTTTL

>AtZF-HD3_AT2G02540.1

MEIASQEDPIPINTSYGNSGGGHGNMNHHHHANSAPSSLNITTSNPLLVSSNSNGLGKNH

DHSHHHHVGYNIMVTNIKKEKPVVIKYKECLKNHAATMGGNAIDGCGEFMPSGEEGSIEA

LTCSVCNCHRNFHRRETEGEEKTFFSPYLNHHQPPPQQRKLMFHHKMIKSPLPQQMIMPI

GVTTAGSNSESEDLMEEEGGGSLTFRQPPPPPSPYSYGHNQKKRFRTKFTQEQKEKMISF

AERVGWKIQRQEESVVQQLCQEIGIRRRVLKVWMHNNKQNLSKKSNNVSNNVDLSAGNND

ITENLASTNP

>AtZF-HD6_AT2G18350.1

MEVREKKDEKMEMTRRKSSALDHHRLPPYTYSQTANKEKPTTKRNGSDPDPDPDLDTNPI

SISHAPRSYARPQTTSPGKARYRECQKNHAASSGGHVVDGCGEFMSSGEEGTVESLLCAA

CDCHRSFHRKEIDGLFVVNFNSFGHSQRPLGSRHVSPIMMSFGGGGGCAAESSTEDLNKF

HQSFSGYGVDQFHHYQPKKRFRTKFNEEQKEKMMEFAEKIGWRMTKLEDDEVNRFCREIK

VKRQVFKVWMHNNKQAAKKKDL

>AtZF-HD16_AT3G28917.1

MRKRQVVLRRASPEEPSRSSSTASSLTVRTVRYGECQKNHAAAVGGYAVDGCREFMASRG

EEGTVAALTCAACGCHRSFHRREIETEVVCDCNSPPSTGN

>AtZF-HD9_AT3G28920.1

MLEVRSMDMTPKSPEPESETPTRIQPAKPISFSNGIIKRHHHHHHNNNKVTYKECLKNHA

AAIGGHALDGCGEFMPSPSSTPSDPTSLKCAACGCHRNFHRRETDDSSAVPPPSLLPSST

TTAAIEYQPHHRHHPPPPLAPPLPRSPNSSSPPPISSSYMLLALSGNNKTAPFSDLNFAA

AANHLSATPGSRKRFRTKFSSNQKEKMHEFADRIGWKIQKRDEDEVRDFCREIGVDKGVL

KVWMHNNKNSFKFSGGGATTVQRNDNGIGGENSNDDGVRGLANDGDGGGGRFESDSGGAD

GGGNVNASSSSS

>AtZF-HD7_AT3G50890.1

MELGGKCNAITTTTMISTEVKPHTDPEPEAKPESDPSMALFPIKKENQKPKTRVDQGAKY

RECQKNHAASTGGHVVDGCCEFMAGGEEGTLGALKCAACNCHRSFHRKEVYGHRNSKQDH

QLMITPAFYSSNSSYKPRVMHPTGEIGRRTSSSSEDMKKILSHRNQNVDGKSLMMMMMRK

KKRVRTKINEEQKEKMKEFAERLGWRMQKKDEEEIDKFCRMVNLRRQVFKVWMHNNKQAM

KRNNSNISE

>AtZF-HD2_AT4G24660.1

MNFEDQEEDMEMSGVNPPCGYDSLSGEGATSSGGGGVGRSKGVGAKIRYRECLKNHAVNI

GGHAVDGCCEFMPSGEDGTLDALKCAACGCHRNFHRKETESIGGRAHRVPTYYNRPPQPH

QPPGYLHLTSPAAPYRPPAASGDEEDTSNPSSSGGTTKRFRTKFTAEQKEKMLAFAERLG

WRIQKHDDVAVEQFCAETGVRRQVLKIWMHNNKNSLGKKP

>AtZF-HD8_AT5G15210.1

MDVIATTTTIVSDLDSRQPEIEAPIRIQPAKPISFSNGKRCHHHHLASEAVAVATYKECL

KNHAAGIGGHALDGCGEFMPSPSFNSNDPASLTCAACGCHRNFHRREEDPSSLSAIVPAI

EFRPHNRHQLPPPPPPHLAGIRSPDDDDSASPPPISSSYMLLALSGGRGGANTAVPMSRK

RFRTKFSQYQKEKMFEFSERVGWRMPKADDVVVKEFCREIGVDKSVFKVWMHNNKISGRS

GARRANGGVVVGGVGDSRQSVVPTNGSFSST

>AtZF-HD10_AT5G39760.1

MMDMTPTITTTTTPTPKSPEPESETPTRIQPAKPISFSNGIIKRHHHHHHPLLFTYKECL

KNHAAALGGHALDGCGEFMPSPSSISSDPTSLKCAACGCHRNFHRRDPDNNNDSSQIPPP

PSTAVEYQPHHRHHPPPPPPPPPPRSPNSASPPPISSSYMLLSLSGTNNNNNNLASFSDL

NFSAGNNHHHHHQHTLHGSRKRFRTKFSQFQKEKMHEFAERVGWKMQKRDEDDVRDFCRQ

IGVDKSVLKVWMHNNKNTFNRRDIAGNEIRQIDNGGGNHTPILAGEINNHNNGHHGVGGG

GELHQSVSSGGGGGGFDSDSGGANGGNVNGSSSS

>AtZF-HD13_AT5G42780.1

MDEIKPKKEENSKRRRNVKPICRETGDHVHYLPTCKTKPKPTRTHHAPPPILDSIFKVTH

KPHYYECRKNHAADIGTTAYDGCGEFVSSTGEEDSLNCAACGCHRNFHREELIPENGGVT

ETVLEVLKISSCQFRRIFCSPYGGGKSEGKKKKKEKESYGGDPIIKDRFGGAEEEEGIVK

RLKTKFTAEQTEKMRDYAEKLRWKVRPERQEEVEEFCVEIGVNRKNFRIWMNNHKDKIII

DE

>AtZF-HD12_AT5G60480.1

MVVLYNECLKNHAVSLGGHALDGCGEFTPKSTTILTDPPSLRCDACGCHRNFHRRSPSDG

FSQHRSPPSPLQLQPLAPVPNLLLSLSSGFFGPSDQEVKNKFTVERDVRKTAMIKKHKRT

KFTAEQKVKMRGFAERAGWKINGWDEKWVREFCSEVGIERKVLKVWIHNNKYFNNGRSRD

TTSSMSLNLKL

>AtZF-HD1_AT5G65410.1

MEFEDNNNNNDEEQEEDMNLHEEEEDDDAVYDSPPLSRVLPKASTESHETTGTTSTGGGG

GFMVVHGGGGSRFRFRECLKNQAVNIGGHAVDGCGEFMPAGIEGTIDALKCAACGCHRNF

HRKELPYFHHAPPQHQPPPPPPGFYRLPAPVSYRPPPSQAPPLQLALPPPQRERSEDPME

TSSAEAGGGIRKRHRTKFTAEQKERMLALAERIGWRIQRQDDEVIQRFCQETGVPRQVLK

VWLHNNKHTLGKSPSPLHHHQAPPPPPPQSSFHHEQDQP

>OsZF-HD5_LOC_Os01g44430.1

MANFYSGVLIFSVLLISLWTVTPVLSHSELDYGRRAKNEKTPNDGNDMSKEIGKGEEQHV

NQEAADAAVVLKTKEEIAKRTAEHIQSTIGSSRVAIHEKEELLEKTAEVMSHMAGEVSDQ

LSKVAKEHTKIAVGSIATALKFKQEVLKQAAQRVKDVSEDVHMATKAKQEILQNVAHDMG

KVAGDMATSMAKMAEVAAGVAGGAAAGVATGIAGGFAGGARVHVSGGIHANIHISASASA

HAKASAAASAGVGAKASKSVSGNVGNNAEEYAGANGNVHGKAKAGISAGFGISAGAKVAA

GIGANAGVGGDAQTNAKAGVGAGIGISGGAKVAGGIGAKAGVGADANANAKAGIGAGVGI

SGGAKVGADIGAKAGVGGNANAKAGVGAGVGISGGAKVGAKIGAKAGVGGDVNAKAGIGA

GVGISGDTKLGADIGAKAGVGADANANAKAGIGAGVGISGGAKVGADIGAKAGVGGDAYT

KAKAGVGAGVGISGGAKVASGIGANAGVGADANANAKAGIGAGVGISGAAKVGADIGAKA

GVGGDMNAKAGIGAGVGISGGAKVGADIGAKAGVGADTNVNAKAGIGAGAGISGGAKVGA

DIGAKAGVGADANAKAGIGAGIGISGGAKVGADIGAKAGVGADANANAKAGIGAGVGISG

GAKVGADIGAKAGVAGDTYTKEKAGVGAGVGISGGAKVGAEIGAKAGVGADANANEKAGI

DAGVGISGRAKVGAGIGAKAGIGANANANAKAGIGAGVGISGGAKVGADIGAKARVGADA

NANAKAGIGAGAGISGGAKVGADIGAKAGVGGGAIAKAGIGAGVGISGGAKVGADIGAKT

GVAGDANAKVGIGAGVGISGGAKVGADIGAKAKVGGDTNAKAGIGAGVGISGGAKVGADI

GAKAEVGADANAKAKASIGAGVGISGGAKVGADIGAKAGVGGDVYTKAKAGVGADVGISG

GAKVAGGIGTNARVGADANANAKAGIGAGVGISGGAKVGADIRAKAGVGGDVNAKAGIGA

GVDISGGAKVGTDIGAKAGVGADANANAKAGIGAVAGISGGAKVGTDIGAKAGVGADANT

KAGIGAGGGISGGAKVGADIGAKAGVGADANANAKDGIGAGAGISGGAKVGADIRAKAGG

GADANANAKDGIGAGAGISRGAKVGADIGAKAGVGADANAKAGIGAGVGISGGAKVGADI

GAKARVGADANANAKAGIGAGASISGGAKVGADIGAKAGVGADANSKAGIGAGVGISGGV

KVGADIGAKAGVGADVNANAKAGIGAGVGISGGAKVGADIGAKAGVGGDAYTKAKAGVGA

GLSEHEEDAGDVGGGCSSPPTPPHRVLTSAAPETIRCRYHECLRNHAAASGGHVVDGCGE

FMPASTEEPLACAACGCHRSFHRRDPSPGRAGAARLPQLHLPASINSRAPPALLLPPAAA

ASKQGLPFPGYGTPSGGTGTTTASSSDERLRPSPVQPRRRSRTTFTREQKEQMLAFAERV

GWRIQRQEEATVEHFCAQVGVRRQALKVWMHNNKHSFKQKQQQENRQEQQQ*

>OsZF-HD7_LOC_Os02g47770.1

MEYKRSSHVEEEEEEEEEEDDEEEDEEEQGHHQYTTAAAQQQLHPQVLGSSASSPSSLMD

SAAFSRPLLPPNLSLVSPSAAAAAAPGGSYLHAAHHHGQGRRVEAPGGESQHHLQRHHEP

ARNGVLGGVAGAHAASTLALVGGGGGGPRGGEGAAGEAPTWRYRECLKNHAARMGAHVLD

GCGEFMSSPGDGAAALACAACGCHRSFHRREPAVVAPASLSLCPASASASAAAGLVSLSP

SATPTGANSSRLMPLLLAPPHMQKRPPVLPVSPASAPAALAESSSEELRPPPLPSSHPHA

HAAAVVAASASAPPGPSKKRFRTKFTAEQKERMREFAHRVGWRIHKPDAAAVDAFCAQVG

VSRRVLKGGDTSTALAAKPPFLRAFAAFFGGQFCLSCF*

>OsZF-HD11_LOC_Os03g50920.1

MEQQQERPREVYRECMRNHAAKLGTYANDGCCEYTPDDGHPAGLLCAACGCHRNFHRKDF

LDGRATAAAGGAGGAGVGVAPMLPAPGGGGPPGYMHMAAMGGAVGGGGGVDGGGGSGGRR

RTRTKFTEEQKARMLRFAERLGWRMPKREPGRAPGDDEVARFCREIGVNRQVFKVWMHNH

KAGGGGGGGGSGGPGAGGGAQTSSSTTRGGGDVGVGLSPAMGGDGEDDEEVRGSEMCM*

>OsZF-HD8_LOC_Os04g35500.1

MMDHLSLVPYEGGSAGGGGGGGKYKECMRNHAAAMGGQAFDGCGEYMPASPDSLKCAACG

CHRSFHRRAAAGIGGGPVFFRPPPPPQPHSHHAALQGFLPSSVPAPAPPPQLALPYHAVP

AAAWHHAAAAAAGRAGSETPPRMDDFGPGSAGGSGSGGGGIFGRKRFRTKFTPEQKERMR

EFAEKQGWRINRNDDGALDRFCVEIGVKRHVLKVWMHNHKNQLASSPTSAAAAAAGVMNP

GAGIGLGTGLGTGISGDGDGDDDDTDDSPPRAAVSSPSPSPISV*

>OsZF-HD6_LOC_Os05g50310.1

MEFRGHDEPVDEMGVAYGRTPPSSSSSPAASASAGNGAGAAEVRYHECLRNHAAAMGGHV

VDGCREFMPMPGDAADALKCAACGCHRSFHRKDDGQQQQQLRLLIPSPPTPRVPLLMPPP

QPQPHPHPQHPYLHPPFPYHHTPSGSGGTTTESSSEERGPPSSSAAAAQGRRKRFRTKFT

PEQKEQMLAFAERVGWRMQKQDEALVEQFCAQVGVRRQVFKVWMHNNKSSIGSSSGGGSR

RQPQEQQSQQQQQQQ*

>OsZF-HD15_LOC_Os06g23030.1

MKLSTFATAAGAAADRSRGGGGKHGVRAASRVGATGGVVAGDMAELQLRRAQPAVGGGET

VYQECPKNHAASLGGHGAGRLRGVHAVVGGEPTDPTSLMCAACGCHCNFHCWLLEGSPPP

PPPLALPAPPMPANVLHGQLHREEETPEVRLPGVDGDESDNNSDGSEYYDERSVSPPSPP

HLPAPVVHQPYYPSAQHMLLSLGSSGQAQRLPL*

>OsZF-HD10_LOC_Os08g34010.1

MEAVVGVKYRPVVFPNGGAAAAAAGKSKATPASATAAVYRECLKNHAASLGGHAVDGCGE

FMPSPAADAADPASLKCAACGCHRNFHRRLPEAPPSPPLLALPPPPPPPPPPPPPPQPQQ

HLPRTAAVAVAPQLLLHGSHQRREQSPETDRVRGPGHHHDDDAAADDDDSEDSEMSDYDD

DRSASPLQAPPPVLSPGYLPSATHMLLSLGSASAPAVAASRPHAAAAAMGPPPPPGAATS

ASRKRFRTKFSPEQKQRMQALSERLGWRLQKRDEAVVDECCREIGVGKGVFKGQLVLPLS

TVITSFVLELIWVCMDNDDDDVLIGVLGGVVF*

>OsZF-HD2_LOC_Os08g37400.1

MDFDDHDEGDGDEEMPPMPLSSGYDAPMQPGLGGGGGGVPKPGGGVGGGGGGGGGGGGGG

ARYRECLKNHAVGIGGHAVDGCGEFMASGEEGSIDALRCAACGCHRNFHRKESESPTGVG

PAEPSAVSPAAISAYGASPHHQFSPYYRTPAGYLHHQQHQMAAAAAAAAAAAAGGYPQRP

LALPSTSHSGRDEGDDMSGMVGPMVIGPMVGMSLGSAGPSGSGSGKKRFRTKFTQEQKDK

MLAFAERLGWRIQKHDEAAVQQFCEEVCVKRHVLKVWMHNNKHTLGKKAP*

>OsZF-HD14_LOC_Os09g24810.1

MMKRLVVLRRREPAVRFSCCGVRYGECRRNHAASTGGHAVDGCREFIAAEDGGGGNSTSA

VGVAAAALKCAACGCHRSFHRRVQVYEVAWDDDCASGDTSSSSPSSSSSLSSE*

>OsZF-HD9_LOC_Os09g24820.1

MEAMDVKYKPLVFPNGAIKKAAKPAAVAPAVGGGGGGETVYRECLKNHAASLGGHALDGC

GEFMPSPAANPADPTSLRCAACGCHRNFHRRLPEGSPPPPPPPALLPAPPMPPHRGEETP

EVRLPGVDGDESDSDSDGSEYDDERSVSPPPPPLAAAVAHQVYYPSAPHMLLSLGSSGQA

QRLPPQVMSPAAAAAPPPGGGGGGMPRKRFRTKFTAEQKQRMQELSERLGWRLQKRDEAI

VDEWCRDIGVGKGVFKVWMHNNKHNYLGGHSARRSASSSSAAAAAAPPFNPPTSPPPPPP

PPPHATDFNINGTATAATAAAAATIAAGNHQENGASSPQSA*

>OsZF-HD1_LOC_Os09g29130.1

MDFDDHDDGDEEMPPMPVSSSYETPPQHGLAGGGMAPKPPGEIGSRVKGPSCGGGRYREC

LKNHAVGIGGHAVDGCGEFMAAGEEGTIDALRCAACNCHRNFHRKESESLAGEGSPFSPA

AVVPYGATPHHQFSPYYRTPAGYLHHHQHHMAAAAAAAAAAAGGYPQRPLALPSTSHSGR

DDGDDLSGMVGPMSAVGPLSGMSLGAGPSGSGSGKKRFRTKFTQEQKDKMLAFAERVGWR

IQKHDEAAVQQFCDEVGVKRHVLKVWMHNNKHTLGKKLP*

>OsZF-HD12_LOC_Os11g03420.1

MGPQQDRSAAKPYANGSTAAAAAAGRKENNKVVRYRECQRNHAASIGGHAVDGCREFMAS

GAEGTAAALLCAACGCHRSFHRREVEAAAAECDCSSDTSSGTGRR*

>OsZF-HD4_LOC_Os11g13930.1

MVSILQLQTRTEASPASSASAAATRIFAVRRQQQEQEGEEEEEEFEFQERMDLSGAQGEL

PIPMHASAAASPFAGMGAHGGAGGGHVVELHRHEHVGNNGQAMAMASPPPTNVAVAAEQE

GSPVAGKKRGGMAVVGGGGGVAVKYRECLKNHAAAIGGNATDGCGEFMPSGEEGSLEALK

CSACGCHRNFHRKEADDLDADSCAAALRAAAGRHHHLLGPALPHHHHKNGGGLLVAGGDP

YGAAYAAARALPPPPPPPPHGHHHHHQIIMPLNMIHTSESDEMDVSGGGGGVGRGGGSSS

SSKKRFRTKFTAEQKARMLEFAERVGWRLQKLDDAMVHHFCQEIGVKRRVLKVWMHNNKH

NLAKKPLPSSPPPPPQIPPMSMPPSPPPPQIPPMSMPPSPPPMPMPMPPSPPQLKLE*

>OsZF-HD13_LOC_Os12g03110.1

MGPQQDRSAAKPYANGSTAAAAAAGRKENNKVVRYRECQRNHAASIGGHAVDGCREFMAS

GADGTAAALLCAACGCHQSFHRREVEAAAAECDCSSDTSSGTGRR*

>OsZF-HD3_LOC_Os12g10630.1

MDLSGAQGELPLPMHAAASPYLGLHHDHHHHHGGGGGGGGMNGRHMSPPTPPAAAEESKA

VVVVSSSATAAARYRECLKNHAAAIGGSATDGCGEFMPGGEEGSLDALRCSACGCHRNFH

RKELDAAAAPPLHHHHHQLLGVGAHPRGHGHHHHHLLVAALPPPTRMVMPLSAMHTSESD

DAAARPGGGAAARKRFRTKFTAEQKARMLGFAEEVGWRLQKLEDAVVQRFCQEVGVKRRV

LKYGSGAPEELKKLDFFWERRGGGGGNPLRNKMKIHLASASTSIRLQLSPTETL*

>SlZF-HD1_Solyc01g014970

MANMLRANQGARAICKPKDLIVYRDCNHSVLPSVSVVDSCQAFASGGPIGSMESFQCQFCHCHQNVHRWLDVNNHQIAPPPAQQQTVTQRDVKPEVSSNTTNANRLGRINAQIFDSLAVSIELAESIMARATENWVN

CNNKDVGNKSKKSWDEHVAEEYEKEQPQILANQLELTLNMRSQNGKNKKIKKDEDEKPDGFELYIKTKSKAIKKLQIAQAENNVFGQSYYPN

>SlZF-HD2_Solyc01g102980

MELPSQDHEDMPIPINSTYGHLIHHDPTPPNNTNHIIPPSMNGPPIDAPPVATAADHHVPFKKIVRYKECLKNHAASMGGNATDGCGEFMPSGEEGTIEALICSACNCHRNFHRKEVEGDQQQLASSCDCFHHVNNRVL

GGGSTKKVYLGHNHHKTSLGPEPFGTIIPTRPPHHQMIMSYNMGSLPNSESEEHDIQDHHHIGGIMGMARPLHHVKKRFRTKFTQEQKDKMLNFAEKVGWKIQKQEEGVVQQFCQEVGVKRRVLKVWMHNNKHSL

AKKNITTNIPNENQLP

>SlZF-HD3_Solyc01g103810

MASNSSSNPNGDITIKYGICLKNHATKFGDYSVDGCREFVKKGDDGTKEAFICANCGCFRDFHRMNSQSLFRLAIHRSRFIHPHVMPHGGGNAPINFHPFMARVMSVQYIRRPVFY

>SlZF-HD4_Solyc01g103820.1

MASNSSSNSNDDMTIKYGICLKNHATKFGDYSVDGCREFVKKGDDGTKEEFICANCGCFRGFHRMNSQSLFRPPILRSCFIHAHVITHGGGHTG

>SlZF-HD5_Solyc01g103830

MASNNKKYFLVKYLECRHNYAARSNGYVLDGCGEFCPTGALETLESFICAACHCHRNFHRKVEVELEDGVESPIISINHPSRGTPLVIIDDPPLQYTVKSRAQFCETSKKNNIDVETKMKRDIGEIKVRKLKRKYNASSS

KRMRLNPYQRERIWIFANEIMRWKWTKSNEQVIPFCDEIGIAPKFLKNWINNTKSRTRPLAKNGHVRNKK

>SlZF-HD6_Solyc01g103840

MASNNNKHFLVKYLECRHNYAARSNGYVLDGYGEFCPTGAPETLEFFICAAWHCHQNFHKKVEVEVENKVESPVISINPSHGTC

>SlZF-HD7_Solyc02g067310

MELTNNNNTTISTITTTTTVKTPEAEIETPTQIQKLKPFPFSNGVLKRKSSFNHNNHHHPVVVIYRECLKNHAASLGGHAVDGCGEFLPSPAANPSDPTSLKCAACGCHRNFHRREPEEPVVIPPPPIATAVLEYQPHHRH

HPPHPPPPLQGEHSSPNSPSPPPISSAYYPASAPHMLLALSAGFSGEKNQNPTSAPLGHSNGRKRFRTKFTPDQKVKMQEFAERVGWKMQKRDEDLVSNFCNEIGVEKGVLKVWMHNNKNTFGKKSDQPNSGSGDGD

NDNDDNHHQNATSA

>SlZF-HD8_Solyc02g067320

MELNINTNTTAAITTVKTPELAETETPSRIQQPKPFSFSNGVLKRKNHHHPVVVVYKECLKNHAANLGTHAVDGCGEFLPIPAANPADPTSLKCAACGCHRNFHRREPEEPPPIATAAIEYQPHHRHHPPPPRGDHGSPN

SPSPPPISSAYYPASAPHMLLALSAGFSGEKNQNLPTSTTPMAVANSNGRKRFRTKFTPDQKIKMLEFAEKVEWKMQKRDEDLVNNFCNEIGVEKGVLKVWMHNNKTTSISGKKLDQPNTDNGHNHQNGNSNYTVN

GFCIVDRNNTTHHHDNTDSEFHIHHESSMNDDNKKENSSFGANNVVVTNGSSSSS

>SlZF-HD9_Solyc02g067330

MRKIQLFENQDDESVTSESSTNSAFTVRIVRYKECQKNHAARVGGHAVDGCREFMPSGEEGTSSAFICAACGCHRNFHRREVETEVASHSLSSSSSSSCVLF

>SlZF-HD10_Solyc02g085160

MEHRGQEKDMGLPNPNPMSYNPSQLNQQESSSSAANKFLTAPNRTTNEHENTIFSPNQTLDQHNITQNSDPDPVRQLSTSSASERNITPVRYKECLKNHAANLGGYVLDGCGEFMPSGEEETLEYLKCAACDCHRNFH

RKETEDESQTPGVHRNNHRIPNQTPPSLPAVPTQQQHHHKYPHSYPRGHMAPVMMSFGGNTGVAAESSSEDLNMFHGGQGVIQPCNFSASKKRFRTKFSQQQKDRMLEFAEKLGWRIQKQDEQEVHQFCNEVGVK

RQVFKVWMHNSKQATKKKQN

>SlZF-HD11_Solyc02g087970

MKKVLRRNDYSRNSTNSSFTMRRVRYVECQRNHAASVGGYVIDGCREYMPEGTTSGTLNCAACGCHRNFHRREVETDVASECTSASSTTK

>SlZF-HD12_Solyc03g061620

MTKRHEDDEENDGSLHTSITIRTVRYRECQRNHAAGVGGYAVDGCREFMPSGEEGTPGALSCAACGCHRNFRRREVETEVASNCSSPS

>SlZF-HD13_Solyc03g098060

MQGDKSNDIYRECLRNHAASLGSYATDGCGEFTLDDTNTSPGGSTSLNCAACGCHRNFHRKFSCGGSYSNNSSRDDREIIAAHDYRLATTEESPAVSERSGKKRFRTKFTGDQKEKMLAFAEKLGWTLQRKDEENET

ERFCREIGVSRKVFKVWMHNHKNNSSSVSSTVTGNNASSLTQ

>SlZF-HD14_Solyc03g116070

MMKKRQVVVRRISSGSSTIRNVRYVECQRNHAANIGGYAVDGCREFMATGDDGTAALTCAACGCHRNFHRREVDGGEVVSESS

>SlZF-HD15_Solyc04g014260

MEFEDQQKQRDEEIAAAPRHNDSLDNSDLTTKMPPSPQLELEPLTAVQLWTNNKPKYKECLKNHAVGIGGHAVDGCGEFLPAGEDGSIDSLKCAACNCHRNFHRKIAPPPIAAAAAGVGGEPVPFVYHSHNQLPTYY

RTLPPPCGYLQYHVAPNQRPLALPSTSGGYREDQEDISHPNYSGGSKKRFRTKFSQVQKDKMQELADKLGWRIQREDEELVQQLCNETGITRQVFKVWMHNNKHTLGKKP

>SlZF-HD16_Solyc04g074990

MEFDDDQEEQEQEIGAVGAENYVTTGNNSGREEGISTSSIGRKSNVRYRECLKNHAVGIGGHALDGCGEFMPAGEEGTMDALKCAACNCHRNFHRKEAEERLGWRMQKQDEALVQQFCAETNVKRHVFKVWMH

NNKHTLGKKP

>SlZF-HD17_Solyc04g080490

MALGGGNEEKEMRISGFQNIDLVSPGGAAPDDIVSAAPHNNSTTGSNKLNKGTSTVRGRYRECLKNHAANIGGNVTDGCGEFMPSGEEGTLEALKCAACNCHRNFHRKEIPNNVGAGVGGGVGGDNNNNAGIMVV

HPLQLPQPLPSPIPSLNHHHHHHHQHGGRSIWTTMPPQPVKMAFGGSGGGSGATDSSSEELNFNTYHHQQATSVPPQQPFMLAKKRFRTKFSQEQKEKMLEFAEKLGWRIPREDDAEVQRFCSQVGVKRQVFKVWM

HNNKNPSSAKKNIIQEDQP

>SlZF-HD18_Solyc05g007580

MELTYSINPTPTPSSTKTPDSEVDTPPLIKPLSFTNGNNHHSHHNQSPPCTAVIYKECLKNHAASIGGHAVDGCGEFMPSPESTPSDPISLKCAACGCHRNFHRREPSDDSSPPAHFIDFRRHIFPPIKRFSPSPTPSPSLSPSL

SPPPLPSLFQPQPVTPTGLKSENPNGRKRFRTKFTAEQKEKMHSFSEKLGWKLQKCDEAAVDEFCNEIGVGKGVLRVWMHNNKNTFGKKDYQISNNSSRDHSFENKNGFNINGTASSNEEEDQHRNNNNDNSTTSNC

ELHLHISTNASSSSS

>SlZF-HD19_Solyc05g018740

MSNNSSSNPSGDMTIKYGICLKNHATNFGDYSVDGCREFVKRGDDGTKEAYICANCGCLRSFHRMNSHSLYHPPILRSRFLHPHIHPHGRENAPIISHPFMSRFVLVQYIRRPVFYNHP

>SlZF-HD20_Solyc05g020000

MTNSSNSNPSGDMTIKYGIYLKNHATNFGDYSVDGCREFVKKGYGGTKEAYICANCGCLRRFHRMNSHSLYPPPILRSHFFHLHVHPHGGENAPIISHPFMSQFVSIQYIRRPVFYNYR

>SlZF-HD21_Solyc05g051420

MSKYKECLKNHAAGIGGHAVDGCGEFMPSGDIFKCAVCNCHRNFHRKDTVHHHHPCGYFPHIMPRRRSLVLPSTSRGGGFREDQELLEMCNPNKNIIGTLLKKRFRTKFSQEHKDKMLRLAEKLGWKLQRHDEGVV

QQLCNEIGIKRHVFKVWLHNNKHTLGKKTNP

>SlZF-HD22_Solyc09g089550

MAAPQRDFNIENQEEPKSKIVYLNYRRVPAVIFRKKYWDWNSMVAVCKLTMVGKFFIPKPKMTKIRASFHAKLSLKGVVKIRSYDSYHVFIDFTAEEDYQSVLLKERVVVAGAIMEVFQWTPEFHDQFREAFGVVEL

ENTKKDEHIGGGNEYGTEESDSVACVPEVTHSDSEDRVASPIKCETSKLPSSVGISCSEFSGLSSAPYRITGRIPSVTYESSSMCSTELVPFRGTYLNHNNLKSYSREWNYGSKPTSEAADLACETLSQPLYALSIVERHEN

QKSRPPIEVQHSTYMGYPTLTFPRRYGESLVSDYKLTLLGNFSYKRPKMKEIRADFKAQNPLSGQVKIRNCSSRQVLILFSNEEDYYTVLYKKAIIVAGALMQISWSSPDFHHEVKQNIHPDFRLSDAKSVNWNTDTSK

LHPSINGLLAPATDEKSMSLQVPAVSQCLSVPSICPPLPLLAHSVSVAGRLCADRLTTSGIYVPQSYPKGIVGIPIFGSNEQAGSGNTFPRPSQALNQQSLAQQHNQLNSYKPNMTVRIAQLQPPSTTSVSDITSSIRYRECL

KNHAASMGGHALDGCGEFMPSGEEGTPGALKCAACNCHQNFHRKEIDDYQPMDDVGSHSRFSQPRNNSSSGSIQNQVLISLPTQQYHHDYSDSCSPRSLVDSLQPYTQPPSPTSGSVYSQQALERIQPSSIRSSYSYEM

VNHNTMQNGKQREYFWSDSSRNTSRDHPSVRNENWNFDMDKPLNNRTPDHIPLEFPTYPSRCQPQTVFTDEFPHLDIINNLLHEEHETGRTLMSNSGSQRLNKGS*

>GlymaZF-HD1_Glyma.01G025000.1

MEFEDQEEQE EELCMGGGAG YDPTQMKIPV AAEPVRSSSS NGGGCGRARY RECLKNHAVG

IGGHALDGCG EFMAAGMEGT LDALKCAACS CHRNFHRKEA DSSAVVSLSG GDPYFLPHHH

HHHHPPPPQF SGYYRHPAGY LHMGGQLRSA VGGTLALPST SGGGGTQSTR EDQEDISNNP

SAGGTGSKKR FRTKFTVEQK DKMLELAEKL GWRIQKHDEA VVQAFCDETG VKRHVLKVWM

HNNKHTLVCY NGGA

>GlymaZF-HD2_Glyma.01G047900.1

MDLTKDTNTL DTNSQTPPQP FTTNGSLKDH HHHHPTTVSP PQQPPSTAVA YKECLKNHAA

SIGGHALDGC GEFMPSSFSN PNEPRSLTCA ACGCHRNFHR RRDTPENHHR SNSRPNFLSF

YHSPPPSRHG AGPSSSPSPS PMSSPSPPPI SHHFPPSSHH FQGPIPAHGL LGLGNEHHHH

MSFNFNSSSH WNPENSGGKK RHRTKFSHEQ KEKMHNFAEK LGWRMQKGDE GLVQDFCKEI

GVSRGVFKVW MHNNKNTSGR KKSLEPGERI NGTGSDSTDN NNNHPYNTNS TNDDTH

>GlymaZF-HD3_Glyma.01G174200.1

MAMRGQQDKE IEIPTTTTLG YNLLPNRDSS SSSSKLSSPT VGERSSSDHD HQTHTLIFNE

TPHHNLYPPP PSLAPPQPQR PTLDPDLSTP IAPTSNPPRT STPSIRYREC LRNHAASMGS

HVVDGCGEFM ASGEEGTPES LRCAACECHR NFHRKEVEGE LQPQSLPQQH VPNYHSYYTN

KHNGHFHYPT PSSSSLHHRL VATTTATPSL VPPVMMAFGG PAESSSEDLI NNTGAQLSVQ

QQAPLTHSSN KKRFRTKFSQ HQKDRMMEFA DKIDWKIQKH NEQEVQHFCT QVGVKRQVFK

VWMHNNKQTS SSKKQQM

>GlymaZF-HD4_Glyma.02G040100.1

MNEYTFGQRK HEGTERETLR EREYETPTTR ISQNLLNSIE KLTKLKEFRE MEFEDQEEQE

EELCMGGGGA GYDPTRMKVP VAAEPVRSTN GGGGRARYRE CLKNHAVGIG GHALDGCGEF

MAAGMEGTLD ALKCAACSCH RNFHRKEADS SAVVAFSGGD PYLIPHHHPP PQFAAYYRHP

AGYLHVAGQQ HRSAVGGTLA LPSTSGGGGT QSTREDQEDI SNNPSAGGTG SKKRFRTKFT

VEQKEKMLEL AEKLGWRIQK QDEAVVQAFC NETGVKRHVL KVWMHNNKHT LGKKP

>GlymaZF-HD5_Glyma.02G056100.1

MDMREQDKVI EMPSTLGYNN SSSGSKLSSP IGERSSDQLP PHQSHTLVFT DPPQTSSHHH

NLYPPSLPPN PLQLPQPHHR PRRDPDPSSI ISPPIISTTP TTAPPQPHTT TTLFRYRECL

KNHAASMGGH VTDGCGEFMP NGEEGTPESL KCAACECHRN FHRKEPHQGV LVESQLQHVL

LNKNNRNINT IIHSPDSHHH LQFPTPHSHL HGGPPVVQPV MLGFGGSGPA ESSSEDLNMF

QTNDHGGGGN NLLLSSVQQQ PPLLSSSSSK KRFRTKFTQQ QKDRMMEFAE KLGWKIQKQD

EQELHQFCSQ VGVRRQVFKV WMHNSKQALK KKQM

>GlymaZF-HD6_Glyma.02G107300.1

MDLTKDTNSQ TPPQPNTTTN GSLKHHHHHP TTVSPPQQPP STTVFYKECL KNHAASIGGH

ALDGCGEFMP SSSSNPNEPR SLTCAACGCH RNFHRRRDTQ ENHHRSNSRP NFISFYHSPP

LSRHGPGLSP TPSPMSSPSP SPPPISHHFP PSSHHFQGPI PAHGLLGLGN ENHHHHMSFN

FNSSSHSTQG NTSGKKRHRT KFSHEQKQKM YNFAEKLGWR MQKAEEGLVQ DFCNEIGVSR

GVFKVWMHNN KNTSARNKSL EPGEKINGTG THSNNNNNHP YNTNSTNDDT H

>GlymaZF-HD7_Glyma.02G180800.1

MEFKQHEETE LRMPAATTSY DDFGIPPSSQ GEEEPTAAAI PVAIPMTPTP PTLAQNNHNE

KYHECLKNHT VKTGVHTLDG CIKFLPLGEE GTLDALKCLT CNCHRNFHRK ETPNYTYLVP

YYRHSSLPLA AYYGEQVGYP HVQGQQCTTL ALPSRSRGIG GAQSSREDME AVSDPTSGAT

PHGGSSKKRF RTRFTQEQKE KMLAFAEKLG WRILKHDESA VQEFCAQTSI QPHVLKVWVN

NNKNTLVTII ATTIRL

>GlymaZF-HD8_Glyma.02G180900.1

MEFKQHEETE LRMPAATASH DDFRIPPSSR GEEEPVVAAI PVAIPVTPTP PTLAQNNNNE

KYHECLKNHT VKNGGHTLDG CITFLPLGEE GTLDALKCLV CNCHQNFHRK ETPNDTYLVP

YYHHSSLPLA VYYGEQVGYP RVQGQQCTSL ALSSRSRGSV SDPTSGAIPH GGSSKKRFKT

RFTQEQKEKM MAFAEKLGWR ILKHDESVMQ EFCSQASIQP HMLKVWVHNN KHTLVWGDSY

FYGRFMR

>GlymaZF-HD9_Glyma.02G211900.1

MVPKKGGKLE SDMEFDEQEE HEEEEEMGIP EPAVAVAAPQ TFDSRSKIGG GEARKSAFGV

AAVRYRECQK NHAVSFGGHA VDGCCEFMAA GDDGMLEGVI CAACNCHRNF HRKEIDGEMS

SFHHRAQPPP PPLHHHHQFS PYYHHRVPQH PTAAGYIHHH LTPPMSQHRP LALPAAASGG

GLSREEEDMS NPSSSGGGGG GGGGGSKKRF RTKFTQEQKD KMLAFAEQLG WRIQKHDESA

VEQFCAEINV KRNVLKVWMH NNKSTLGKKP

>GlymaZF-HD10_Glyma.02G211900.2

MVPKKGGKLE SDMEFDEQEE HEEEEEMGIP EPAVAVAAPQ TFDSRSKIGG GEARKSAFGV

AAVRYRECQK NHAVSFGGHA VDGCCEFMAA GDDGMLEGVI CAACNCHRNF HRKEIDGEMS

SFHHRAQPPP PPLHHHHQFS PYYHHRVPQH PTAAGYIHHH LTPPMSQHRP LALPAAASGG

GLSREEEDMS NPSSSGGGGG GGGGGSKKRF RTKFTQEQKD KMLAFAEQLG WRIQKHDESA

VEQFCAEINV KRNVLKVWMH NNKSTLG

>GlymaZF-HD11_Glyma.02G211900.3

MEFDEQEEHE EEEEMGIPEP AVAVAAPQTF DSRSKIGGGE ARKSAFGVAA VRYRECQKNH

AVSFGGHAVD GCCEFMAAGD DGMLEGVICA ACNCHRNFHR KEIDGEMSSF HHRAQPPPPP

LHHHHQFSPY YHHRVPQHPT AAGYIHHHLT PPMSQHRPLA LPAAASGGGL SREEEDMSNP

SSSGGGGGGG GGGSKKRFRT KFTQEQKDKM LAFAEQLGWR IQKHDESAVE QFCAEINVKR

NVLKVWMHNN KSTLG

>GlymaZF-HD12_Glyma.04G093300.1

MEFDEHEDQE EEEEEEMGFS VAAASYDSLG NAAVRSKMSG GEGVAVTGNS GRKGTLRYRE

CQKNHAVSIG GQAVDGCCEF LAAGEEGTLE AVICAACNCH RNFHRKEIDG ETSPYRQRSQ

PQPQPLHPQY HHQFSPYYHR APPPSAAGYL HHHLVTPPVS QHRPLALPPL ASGGVFSREE

EDMSNPSSSG GGGGFSGGGG GSGSGTKKRF RTKFTQEQKD KMLAFAEELG WRIQKHDEVA

VEQFCAETCV KRHVLKVWMH NNKHTLANSE QRSAGNGSNH LNSTTIIENS QLS

>GlymaZF-HD13_Glyma.04G169900.1

MKKRQVVVKS VANTSSSVMR NIRYGECQKN HAANIGGYAV DGCREFMAST GEGAGGALTC

AACGCHRNFH RREVNTEVVC EYSPPNSGR

>GlymaZF-HD14_Glyma.05G026900.1

MKKRQVVVKR DYATSSPAVG NIRYGECQKN HAANTGGYAV DGCREFMASA GEGTNAALTC

AACGCHRNFH KREVLHGVN

>GlymaZF-HD15_Glyma.05G248200.1

MEGGSGGERN SSVYRECLRN HAASLGSYAT DGCGEYTVDG AGGLQCAACG CHRNFHRKVK

YLAAAESPPT EYGGSNSKKR FRSKFTEDQK EKMLGFAEKL GWKLQRRDLD DEIERFCRSV

GVSRQVFKVW MHNHKNSSSS SSTAANVSSL TQ

>GlymaZF-HD16_Glyma.06G095200.1

MEFDEHEDQE QEEEEEEEEM GFSVAAAASY DSLGNAAVRS KISGGDGVAA TVNSGRKGTV

RYRECQKNHA VSIGGHAVDG CCEFLAAGEE GTLEAVICAA CNCHRNFHRK EIDGETSPYQ

HRSQPQPQPL HPQYHHQFSP YYHRAPPPSA AGYLHHHLVT PPVSQHRPLA LPPLASGGVF

SREEEDMSNP SSSGGGGGGG FSGGGGSGSG TKKRFRTKFT QEQKDKMLAF AEKLGWRIQK

HDEAAVEQFC AETCIKRHVL KVWMHNNKHT LANSEQHSAG NGSNHLNSTT IIENSQFS

>GlymaZF-HD17_Glyma.06G193700.1

MKKRQVVVKS VANTSSSVMR NIRYGECQKN HAANIGGYAV DGCREFMAST GEGAGGALTC

AACGCHRNFH RREVNTEVVC EYSPPNSGR

>GlymaZF-HD18_Glyma.07G107200.1

MPISSLSISM STQYIHQINI YIKTYTTPSL KVTQKPHKQE RKMENSSSSN YLYRECLRNH

AASLGSYATD GCGEFTLDAD SVSSPSLQCM ACGCHRNFHR KVTCPVVEGP QVVTGGSGDM

MEYSGGEGRM EMGERSGGGS SKKRFRTKFS AEQKEKMLGF AEKLGWKLQR KEVDDEIERF

CKSVGVTRQV FKVWMHNHKN NSNSSTNSSV NLSSLTQS

>GlymaZF-HD19_Glyma.07G230200.1

MDLTSISTHN TNTTQTLDAA NTTTKTTPPT PIPTTTPKSL SFANGTLKRH PTTTVAQPPS

MVVSYKECLK NHAASIGGHA LDGCGEFMLS SSTVPSDPRS LKCAACGCHR NFHRREPQEN

NLNNNLKNNN PTFLNCIYTL SAPAPLSHRA MSQSTSPSLS SSPSHSQSPI SSPSPPPLSH

VPPYHASAPH MLLALGSAYS AEPSDEHHHH QQQQHKSFNL SMMRSENPTK KRYRTKFSKE

QKEKMHSFSE KLGWRMQKGD DGLVQEFCND IGVSRGVFKV WMHNNKNTFR KKSEDGNGNA

NAPQSNDDDN ANGGGGGFDS DINNPYNPNS NNNDIHMKEV DDACVNNVVH VSLNEMSS*

>GlymaZF-HD20_Glyma.07G271900.1

MELSSQEGEI PIPINSSTTY GHGNGHGHGL MIHHDHNHII SSTAPSNGIP TMQQEEDHGL

GSYKKVVRYR ECLKNHAAAM GGNATDGCGE FMPSGEEGTI EALNCSACHC HRNFHRKEVE

GEPSCDYHHL NINRRRHILG PHKNLLPPEA LGYPTAARSV PPHQMIMPYN IGGIGHHLPS

ESDEQEDGGG GGGMVQLSSR PISSQQQLVK KRFRTKFSQE QKDKMLNFAE KVGWKIQKQE

ESVVQQFCQE IGVKRRVLKV WMHNNKHNLA KKNPPTTAAP PPP

>GlymaZF-HD21_Glyma.08G056700.1

MEGGSGGGER EKWNSVSVYR ECLRNHAASL GSYATDGCGE FTVDGAGGLQ CAACGCHRNF

HRKVKYPVAE HEEYGGKKRF RSKFTADQKE KMLGFAEKLG WKLQRKDLND EIERFCRSVG

VSRQVFKVWM HNHKNSSSSS SSTPANVSSL TQ

>GlymaZF-HD22_Glyma.08G189000.1

MSDINPNQPL PMDEVVTYKE CWHNHAAARG RVIYDGCLEY LVDQDNLICA CCGCHRNFHR

KHTIFNGIPE TQTLDRVQGM RPKRKKRTTF SSEQRNKLIC FAESVGWKPR KDKKNEIQSF

CSEMGMTRRM FLIWLSNNRH RAIKKA

>GlymaZF-HD23_Glyma.08G280900.1

YQECLKNHVV SIGGHAIDGC IEFLPGGEEG TLDALKCAAC NCHRNLHRKE THDTYSVPFR

HHHHPLLPPP VPLAAYYRAL PGYLHMTGHQ CAMLAHPSLS GRGGPQPPWE DLEDSDPTSG

ATTHDGSGSS SKKRFRTKFT QHQKDKMLVF AEKLGWRMQK NDESIVQEFC SEIGVQRHLL

KVWMHNNKHT LGKKP

>GlymaZF-HD24_Glyma.08G281000.1

GKGKYQECLK NHGVSIGKHI IDGCIEFLPG GEEGTLEALK CVVCSCHRNF HRKETHDTYS

VPFHHHHPPL PPPVPFAAYY RAPPGYPHMT GHQRAMLAHP SLSGGGGPQP PLEDLEDSDP

TSGATTHDGS GSSSKKRFRT KFTQHQKDKM LVFAEKLGWR MQKNDDNVVQ EFCSEIGVQR

HVLKVWMHNN KHTLGKKP

>GlymaZF-HD25_Glyma.08G281100.1

GKGKYQECLK NHGVSIGKHI IDGCIEFLPG GEEGTLEALK CIVCSCHRNF HRKETHDTYS

VPFHHHHPPL PPPVPSAAYY RAPPGYPHMT GHQRAMLAHP SLSGGGGPQP PLEDLEDSDP

TSGATTHDGS GSSSKKRFRT KFTQHQKDKM LVFAEKLGWR MQKNDDSAVQ EFCSEIGVQR

HVLKVWMHNN KHTLGKKP

>GlymaZF-HD26_Glyma.08G281200.1

MEFQKHHEEA ELGLPIAVAA TSYEEFGMPL NHGEQEPVVE VIPMAVPMAV PVAPPTNIVA

QNSGKGKYQE CLKNHGVSIG KHIIDGCIEF LPGGQEGTLE ALKCVVCNCH RNFHRKETHD

TYSVPFHHHH PPLPPPVPFA AYYRTPPGYP HMTGHQRAML AHPSLSGGGG PQPPLEDLED

SDPTSGATTH DGSGSSSKKR FRTKFTQHQK DKMLVFAEKL GWRMQKNDDS VVQEFCSEIG

VQRHVLKVWM HNNKHTLGKK P*

>GlymaZF-HD27_Glyma.09G170500.1

MSILVTLHIN VHTIHQINIL KPTLHYTTLH PLSHSHKTTQ ERKKERHMEN SSSSSSCSNY

LYRECLRNHA ASLGSYATDG CGEFTLDVDS VSSPSLQCAA CGCHRNFHRK VTCPAVEGGL

QAVTGGSGDM MEYSGGGDVG RITEMGERSG GSKKRFRTKF SAEQKEKMLG FAEKLGWKLQ

RKEVDDEIER FCKSVGVTRQ VFKVWMHNHK NNSNTSTNSS ANLSSLTQS

>GlymaZF-HD28_Glyma.09G238400.1

MKQKHRPGSP PPSLPQRQKP HLSRMVSSNA PTTTTTSTTL SLRWLPTKNA SKITPPHLGG

HALDGCGEFM PSPTATAADP SSIKCAACGC HRNFHRREPE EPPIASTTTH VIEYQPHHRH

HPPPPLHAAA VATRSPNSAS PPPISSSYYP SAPHMLLALS AGLAAPPEST AAPAAAPTRK

RFRTKFSQEQ KEKMHKFAEK VGWKIQKRDE DLIHEICNEV GVDRSVLKVW MHNNKNTFAK

KDNNNIVTSN DINITTKNNI STDNDANGSD VKVFENPNND GGEDRAEDPS SNHYDGVNVV

GANGSSSS

>GlymaZF-HD29_Glyma.09G238500.1

MRRMTLMGRN GPQRCPNNAL VTIVRYVRYR DCRRNHVCHL GGHTVDGCTE FIPSGSEGTD

TALICAACGC HRNFHRREEY SQLVCGCTSH PSTSGA

>GlymaZF-HD30_Glyma.09G238600.1

MRRVILRRDG SRRCSNNSPV TIVRQVKYGK CCRNLACRIG GHVVDGCTEF VASGAEGTRE

AMTCATCGCH RNFHMKEEIT QVLCACSSHP TTRTVGD

>GlymaZF-HD31_Glyma.09G238700.1

MPRRCPHNTI HRECRRNYAC RVGGYILDGC RQFVASGAEG TAAAMTCATC GCHKNFHRRE

ELPHVVCGCN NEA

>GlymaZF-HD32_Glyma.11G068800.1

MAMRGQQDKE IEIPTTTTLG YNLPNRDSSS SSSKLSSPTV GERSSSDHDQ QHHQPTHQTR

TLIFNETPHH NLYPPPPPPP PALAPHRPTS DPDLSTPIAP TSNPSRTAPP QTTTTTTSTP

SIRYRECLRN HAASMGSHVV DGCGEFMASG EEGTPESLRC AACECHRNFH RKEVEGELRP

QPQPQPQTHV PNYHSYYTNK HNGHLHYPTP SSSSLHHRLV TPTSLVSPVM MAFGGPAESS

SEDLNMFQSN TGGAQLISVQ QHAPLLSSSK KRFRTKFSQH QKDRMMEFAD KIDWKIHKHN

EQEVQQFCSQ VGVKRQVFKV WMHNNKQTTS SKKQQI

>GlymaZF-HD33_Glyma.11G068800.2

MAMRGQQDKE IEIPTTTTLG YNLPNRDSSS SSSKLSSPTV GERSSSDHDQ QHHQPTHQTR

TLIFNETPHH NLYPPPPPPP PALAPHRPTS DPDLSTPIAP TSNPSRTAPP QTTTTTTSTP

SIRYRECLRN HAASMGSHVV DGCGEFMASG EEGTPESLRC AACECHRNFH RKEVEGELRP

QPQPQPQTHV PNYHSYYTNK HNGHLHYPTP SSSSLHHRLV TPTSLVSPVM MAFGGPAESS

SEDLNMFQSN TGGAQLISVQ QHAPLLSSSK KRFRTKFSQH QKDRMMEFAD KIDWKIHKHN

EQEVQQFCSQ VGVKRQVFKV WMHNNKQTTS SKKQQM

>GlymaZF-HD34_Glyma.12G165800.1

MEMRPVTRCI KTILEVDTYT HAPPSPFYLS LTPSMRMHTH PSISINLLLN FTMSNININH

PLPIDEVVTY KECLHNHSAA LGHVTYDGCV KYIAGEDALL CACCGCHRNF HHKNTIFTAE

PQTQTPDQVQ EMRSKRKKRT TFSSEHKNKL IRFAESVGWK PRKEKKDEIE SFCSEMGITR

RMFIVWLINN RHRAINNA

>GlymaZF-HD35_Glyma.12G168600.1

MHQNHIRSGH VHYVPPSPFY LSLSPLVQMH THPSISINLL LNFTMSDINI NHPLPTHEVV

TYKECLHNHS AALDHVTYDG CVKYIAGEDA LLCACCGCRR NFHHKNTIFT AEPQTQTPDQ

VQEMRSKRKK RTTFSSEQKN KLIRFAESVG WKPRKEKKDE IESFCSEMGI TRRKFVVWLS

NNRHQAINDA

>GlymaZF-HD36_Glyma.12G168700.1

MHTHPSISIN LLLNFTMSDI NINHPLPTHE VVTYKECLHN HSTTLGHVTY DGCVKYIVGE

DALLCACCGS HRNFHHKNTI FIAEPQTQTP DQVQEMRSKR KKRTTFSSEQ KNKLIRFAES

VGWKPRKEKK DEIESFCSEM GITRRMFVVW LSNNRHRAIN NA

>GlymaZF-HD37_Glyma.13G062900.1

MEIITPTATA TNATAKSPEP DIETMTRIHQ NPNPNPKPVS FSNGVLKRHH VNHRAAVVYK

ECLKNHVASL GGHALDGCGE FMPSPAATAD DPSSIKCAAC GCHRNFHRRE PEESPISPAT

HHVLEYRPHH RHHPPPPHRS PNSASPPPIS SYPSAPHMLL ALSGGAGLSV APENTAAPAP

PHHSRKRFRT KFTQEQKEKM HEFADKVGWK MQRRDEEMVM EFCNEIGVDR GVLKVWMHNN

KNTFAKKDNL NGNSIGNVAI VSANGVAPIG AVGSATVRSS VHVHEHEHGG INGNGSGDNN

HNPLGSRDVN EYENDSGTNG GGGTNGSSSS S

>GlymaZF-HD38_Glyma.13G063100.1

MRKRQVVVRR EEPQRSARTV KYGECQKNHA ANVGGYAVDG CREFMASGAT GEGTSAALTC

AACGCHRNFH KRQETEVVNH II

>GlymaZF-HD39_Glyma.14G179600.1

MEFDEQEEHE EEEMVMPEPA AVSAPPSYDS LGNSGAMSKL GGGEGRKTAL GAAAAAVRYR

ECQKNHAVSF GGHAVDGCCE FMAAGEDGTL EAVICAACNC HRNFHRKEID GEITSFHYRA

QPPPPPMHHH HQFSPYYHHR VPQHPAAAGY LHHHLTPPMS QHRPLALPAA ASGGGLSREE

EDMSNPSSSG GGGGGSKKRF RTKFTQEQKD KMLAFAEQLG WRIQKHDESA VEQFCAETNV

KRNVLKVWMH NNKSTLGKKP

>GlymaZF-HD40_Glyma.16G101400.1

MSFGGHAAND CCEFLAARED DTLETVICVA CNYHRNFHCK EIDDEITSIH HRHNPLVLLA

IASDGSLSHE EELAWSSQRV VQPTTSYTSL FSENDKQTKF CRTNRNRNHY LLIFECC

>GlymaZF-HD41_Glyma.16G138700.1

MDMREQDKVI EMPSTLDYNN SSSSPKLSSP IGERCDQLPP HQSHTLVFTD PPQTSSHHHN

LYPPSLPPNP LQLPQPHHRP RRDPDPSPII SPPRTTPPQP QPHTTTTLFR YRECLKNHAA

SMGGHVTDGC GEFMPNGEEG TPESFKCAAC ECHRNFHRKE PHQGVVLESQ LLQHVLNKNS

RNINILHSPH SHHVLHGVVG GPVQPVMLGF GGSGPAESSS EDLNMFQTLD HRGGGNLLSS

SVQQPPLSSS SSKKRFRTKF TQQQKDRMME FAEKLGWKIQ KQDEQELHQF CSQVGVRRQV

FKVWMHNSKQ AMKKKQLNIL LESVESYEE

>GlymaZF-HD42_Glyma.16G138700.2

MDMREQDKVI EMPSTLDYNN SSSSPKLSSP IGERCDQLPP HQSHTLVFTD PPQTSSHHHN

LYPPSLPPNP LQLPQPHHRP RRDPDPSPII SPPRTTPPQP QPHTTTTLFR YRECLKNHAA

SMGGHVTDGC GEFMPNGEEG TPESFKCAAC ECHRNFHRKE PHQGVVLESQ LLQHVLNKNS

RNINILHSPH SHHVLHGVVG GPVQPVMLGF GGSGPAESSS EDLNMFQTLD HRGGGNLLSS

SVQQPPLSSS SSKKRFRTKF TQQQKDRMME FAEKLGWKIQ KQDEQELHQF CSQVGVRRQV

FKVWMHNSKQ AMKKKQLNIL LESVESYEE

>GlymaZF-HD43_Glyma.16G138700.3

MDMREQDKVI EMPSTLDYNN SSSSPKLSSP IGERCDQLPP HQSHTLVFTD PPQTSSHHHN

LYPPSLPPNP LQLPQPHHRP RRDPDPSPII SPPRTTPPQP QPHTTTTLFR YRECLKNHAA

SMGGHVTDGC GEFMPNGEEG TPESFKCAAC ECHRNFHRKE PHQGVVLESQ LLQHVLNKNS

RNINILHSPH SHHVLHGVVG GPVQPVMLGF GGSGPAESSS EDLNMFQTLD HRGGGNLLSS

SVQQPPLSSS SSKKRFRTKF TQQQKDRMME FAEKLGWKIQ KQDEQELHQF CSQVGVRRQV

FKVWMHNSKQ AMKKKQLFCM QKHSPRIC

>GlymaZF-HD44_Glyma.17G002200.1

MELSSQEGEI PIPINSSTTY GHGNGHGHGH GHGLMIHHDH NHIISSSAPS NGIPTTMQQQ

EEEEEEEDRY KKVVRYRECL KNHAAAMGGN ATDGCGEFMP SGKEGSIEAL NCSACHCHRN

FHRKEVEGEP QHHLNINRRR LILGPHPEAL GYPTAAARSV PPHQMIMPYN IGIGHHLPSE

SDEQEDAAAG AGMVQLSSRP SSAQLVKKRF RTKFSQDQKD KMLNFAEKVG WKIQKQEESV

VQHFCQEIGV KRRVLKVWMH NNKHNLAKKI NPPTTTAPPP PPPPPP

>GlymaZF-HD45_Glyma.17G100000.1

MKKRQVVVKR DYATSSPAVG NIRYGECQKN HAANTGGYAV DGCREFMASA CEGTNAALTC

AACGCHRNFH KREVLHGVN

>GlymaZF-HD46_Glyma.18G145400.1

MEFQEHHVEA ELGMPAAAAS YEEFGMPPNH GQEEPVVAAV PVPPPANTMA RNSGKGKYQE

CLKNHAVGIG GHALDGCAEF LPAGEEGTLD ALKCAACNCH RNFHRKETPD GTYLLPFHHR

HQPPPPPFAP YYRAPAGYLH MTGPQHATLA LPSTSGGGGT QSPREDQGDL SDPPTSGATT

HGGSSSKKRF RTKFTQQQKD KMLAFAEKLG WRIQKHDEGV VQEFCSETGV QRHVLKVWMH

NNKHTLGKKP

>GlymaZF-HD47_Glyma.18G257200.1

MPAQFLVTNV KYHECRWNHA CRDGGYILDG CREFVASGAE GTDSAMTCAT CGCHRNYHRR

EDFCDCPSHP AANGA

>GlymaZF-HD48_Glyma.18G257300.1

MWRVILHSDG TVRCWNNSPV TIVKHVKYRE CCRNLACRIG GHIVDRCSEF VAYGAEGTSA

AMTCATCGCH RNFHRKEEQI QLVCTCSSHP TTGTGGGA

>ZF-HD49_Glyma.18G259200.1

MKRVILRRDG RTRYSNNSLV TIVRHVRYIA RIVDGYREFV ASGAEGTGGA MTCATCDCHR

NFHRKEEQTQ MVCACSSHPT TQIRRHSN

>GlymaZF-HD50_Glyma.18G259300.1

MDITPTTTII NTTTTPSAAK SPEHETETPT RITTPITPTT TKTLSFSNGV LKRHHHHHLH

HPMPAVVTYK ECLKNHAANL GGLALDGCGE FMPSPTATAA DPSSIKCAAC GCHRNFHRRE

PEDPPITSNT THVIEYQPHH RHHPPPPLHA AAAARSPNSA SPPPISSSYY PSAPHMLLAL

SAGLAAPPES TAAPSAALSR KRFRTKFSQE QKEKMHKFAE KVGWKIQKRD EDLIHEFCNE

VGVDRSVLKV WMHNNKNTFA KKDNNIVISN DINITTSNNI NTSIIDDNDA NGGDVKVFEN

PNNNHGSGSG GGEDPAEDPS RNHYGGVNVG ANGSSSSS

>GlymaZF-HD51_Glyma.19G022200.1

MRKRQVVVRR EEPQRSGVRA VKYGECQKNH AANVGGYAVD GCREFMASGS GSGGGSGGGE

GTSAALTCAA CGCHRNFHKR QEAEVVSECS SPTSNGT

>GlymaZF-HD52_Glyma.20G036500.1

MDLTSSTTHN TNTTQTLEAA NTITITTKAT PPIPTTTLKS LSFTNGTLKR HPITTVAPPP

SMVVSYKECL KNHAASIGGH ALDGCGEFMP SSSTVPSDPR SLKCAACGCH RNFHRREPQE

NNLNNNLKNN NPTFLNCIYS TPSAPAPLSH RAMSQSTSPS LSSSPSHSPS PISSPSPPPL

SHLVR

>GlymaZF-HD53_Glyma.20G070300.1

MTPTPPTLAQ KNDNEKYHEC LKNHTIKTGV HTLDGCIKFL PLGEEGTLDA LKCLVCNCHR

NFHRKETPND TYLVPYHRHS PLPLAAYYGE QVGYPHVQGQ QCTTLALPSR SRGSGGAQSS

REDMEAVSDP TSGATPHGGS SKKRFRTRFT QEQKGKMLAF AEKLGWRILK HDESVVQEFC

AQTSIQPRVL KVWVHNNKHT LSKKL

>GlymaZF-HD54_Glyma.20G075300.1

MEFKHHEETE LRMPAATTSY DDFGIPPSSQ GEEEPVAAAI PVAIPMTPTP PTLAQNNDNE

KYHECLKNHT IKTGVHTLDG CIKFLPLGEE GTLDALKCLM CNCHRNFHRK ETPNYTYLVP

YYRHSPLPLA AYYGEQVGYP HVQGQQCTTL ALPSRSRGSG GAQSSREDIE AVSDPTSGAT

PHGGSSKKRF RTRFTQEQKE KMLAFAEKLG WRILKHDESA VQEFCAETSI QPHVLKVWVN

NNKNTLVTII ATTIRL

>GlymaZF-HD55_Glyma.20G075400.1

MEFKQHEETE LRMPTPTASH DDFGIPPSSQ GEEELVAAVI PVVIPVTPTP PTLAQNNDNE

KYHECLKNHT IKTGVHTLDG CIKFLPLGEE GTLDALKCLV CNCHRNFHRK ETPNDTYLVP

YYYHHSSLPL AAYYGEQVGY PRVQGQQCTT LALPSRSRGS GGAQSSREDM EAVSDPTSGA

TPHGGSNKKR FRTRFTQEQK EKMLAFVEKL GWRILKHDDS VVQEFCAQTS IQPHVLKVWV

HNNKHTLVTI IATTIRL

>GlymaZF-HD56_Glyma.20G075600.1

MEFKHHEKTE LSMPAATASH NDFGIPPSSQ GEEEPVAAAI PVAIPVTPTP PTLAQNNDNE

KYHECLKNHT VKTGVHTLDG CIKFLPLGEE GTLDALKCLV CNYHRNFHRK ETPNDTYLVP

YYHHSPLPLA AYYGEQMGYP RVQGQQCTTL ALPSRSRGSG GAQSSREDME AVSDPTSATP

HGGSSKKRFR TRFTLEQKEK MLAFAEKLGW RILKNDESVV QEFCAQTSIL PHVLKVWVHN

NMHTLVTIIA TTIRL

>GlymaZF-HD57_Glyma.20G075800.1

MEFKQHVETE LRMPVASASH EDFGIPPSSQ GEEEPVAAAI PVAIPMTPTP PTMSQNNNNE

KYHECLKNHT VKIGSHTLDG CIKFLPLGKE GTLDALKCIV CNYHRNFHRK ETPNDTYPES

YHHHPPLPFT AYYRAPVGYP HVQGQQCTTL ALPSRFTQEQ KEKMLAFGEK LGWRIHKHNE

SVVQEFCAQT SVQPHVLKVW MHNNKHTLGK KS

>GlymaZF-HD58_Glyma.20G214300.1

MEDKKIIIRY KECLKNHAAA IGGNATDGCG EFMAAGEEGT LEALKCSACN CHRNFHRKEI

ESSDSNAIPL MIIPDTTQII RPILAHLSPN KSGSISPSDL SDEKENEDGM MIKEVENPNE

KVKKRFRTKF TQEQKEKMLA FAERAGWRIQ KLDESLVQKF CQEIGIKRRV LKVWMHNNKN

TFAKRNPSTS

>BraZF-HD1_Bra003759

MDMRSHEMIERRRDDNGNNGVGNINSIITNEDNCNGNNNTRVSSNSQTLVPHQSKSSPSF

SISTVRYRECLKNHAASVGGSVYDGCGEFMPSGEEGTLEALRCAACDCHRNFHRKENDGV

GSSDGSSHHRHHHHHHQYGGGRRPPPRNMMLNPLMLPPPPSYAPLHHHKYGMSPPGGGGM

VTPMSFPYGGGGGGAESSSEDLNMYGQSSGENGGGVTPGQTAFSMKRFRTKFTAEQKEKM

MEFAEKLGWRMNKQDEEDLKRFCGEIGVKRQVFKVWMHNNKNNARKQPTPTTTT

>BraZF-HD2_Bra003791

MKKRQVVIKQRSRNSNTSSSWTTTSSSATSSIRYVECQKNHAANIGGYAVDGCREFMAAG

VEGTDDSLRCAACGCHRNFHRKEVNTEVVCEYSPPNA

>BraZF-HD3_Bra004399

MGVCYKECLKNHAANLGGHALDGCGEFMPTPTATPTDPSSLRCAACGCHRNFHRRDPSDH

LNFLPSHPTSSPSGTESPPSQSLHNVASPVPCSYYTSAPHHMLLSLSSGFPGPADQDPAA

VRSENSSKGGMRKRTRTKFTAEQKIKMRAFAEKAGWKINGCDEKSVREFCSENGIERGVL

KVWMHNNKYSLLNGKNREILSMEDQPRLCLNTQSCNNGGEDGDNVGGSSSS

>BraZF-HD4_Bra006657

MGFLYNECLKNQAVSFGGYALDGCGEFMPKSTTILTDPPSLSCDACGCHRNFHRRDPSNV

FIHRTNSTPPPPPLQPVASTQHLLLSLSGSGFSGPSDQDMGKISTVDRKRKRTKFTVKQK

VMMRAFAERAGWKINGCDDEYVREFCREVGVEREVLKVWIHNNKYFANGRNRNTTSSMFQ

KL

>BraZF-HD5_Bra007869

MGICYKECLKNHAANLGGHALDGCGEFMPTPTATPTDPSSLRCAACGCHRNFHRRDPCDH

LNFLPVHPTSSPSGTESPPSQSLHHVASPVPCSYYASAPHHVHLSLSSGFPGRSDQDPTA

VRSENSSKGAMRKRTRTKFTPEQKMKMRAFAEKAGWKINGCDEKSVRGFCGEIGIGRGVL

KVWMHNNKYSLLNGKNSEILSMDHPRLCLNTHSCNNGGEDGDTVDGSSSS

>BraZF-HD6_Bra008185

MDMRSHEMIERRSDDNGNNGAGNISSIITNEDNCNGNNNNNTRVSCNSQTLDHHQSKSSS

ISAAAKTTVRYRECLKNHAANVGGSVHDGCGEFMPSGEEGTLEALRCAACDCHRNFHRKE

VDGVGSSAHHHRHHHQYGGGGGRRPPPPNMMLNPLMLPPPPNYAQMHHHKYGMSPPSGMV

TPMSVAYGGGGGGAESSSEDLNMYGQSSGEHGGGATVGQMGFSKKRFRTKFTTEQKERMM

EFAEKLGWRMNKQDEEELKRFCNDIGVKRQVFKVWMHNNKSNAKKPTTTTPGTL

>BraZF-HD7_Bra008712

MDVIATSATIVSHLDSQQPMTQAPTRIQPAKPISFSNGKHHHDHQHHASQVVVAAYKECL

KNHAAGIGGHALDGCGEFIPSPTFNTSDPTSLTCDACGCHRNFHRREDDLSAVSAAVPRI

EFRPHNRHQLPPPPLPPVGVGSQDDDDPASPPPISSSYMLLALSGRATAAPVSRKRFRTK

FSEFQKGKMFEFAERVGWRMPKADDVDVVEFCREIGVERSVFKVWMHNNKTPGRGGGARR

ANGDGGVGDDRENVPTNGSFAST

>BraZF-HD8_Bra010485

MNFEEQEEEMEMSGVHPSGGYDSLSGEGATSSGGGGGGRRTSVGEKTRYRECLKNHAVNI

AGHAVDGCCEFMHSGEDGSLDALKCAACCCHRNFHRKETEMISGRAHIVPTYFNRPPQLP

PPGYRQPAASADEEDTSNPSSSGGTKAKRFRTKFTAEQKEKMFAFAERLGWRMQKHDDVA

VEQFCGETGIRRQVIKIWMHNNKNSLGKKP

>BraZF-HD9_Bra012882

MEHGGNCNAITSTTITLKAKPNTNPDPKTKPGSDSFLIKKENQKPRTRSNQAAKYEECQK

NHAALTGGHVVDGCCEFMPGGEEVTSGALKCAACNCPRSFHRKEVYGHRNSTQEELISPT

FYRSGNSYKAIQPRGVYPTGEIGRRTSSSSEDMKKILNQNSDGKGLMMITRKKKRVRTKI

NEGQKKKMKEFAERLGWSIQKKDEEEIDKFCRTVNLRRQVYSHVQELVNLLLSIIIGDGE

KLKHQHHYKGSRVSRSRSKSSSSLR

>BraZF-HD10_Bra013822

MNFEEQEEEMEMSGANPTGGYDSLSGEGATSSGGGGGGGSRKTVGGSKVRYRECLKNHAV

NIGGHAVDGCCEFMPSGEDGSLDALKCAACGCHRNFHRKETEVMSGRAHRVPTYYNRPPQ

LPPPGYLHLTSPAAAGQPYRPPAASGDEEDTSNPSSSGGTRAKRFRTKFTAEQKEKMLAF

AERLGWRIQKHDDATVEQFCAETGVRRQVLKIWMHNNKNSLGKKP

>BraZF-HD11_Bra015857

MDMRSHEMVERRRDDNGNNGVGSIANEDNVNGNNNNNTRVSSQTLDHHQSKSPSSFVISA

AAKTAVRYRECLKNHAANVGGSVHDGCGEFMPSGEEGTIEALRCAACDCHRNFHRKEVDG

VGSSDVIAHHHRHHHHQYGGGGGGGRRPPPPNMMLNPLMLPPPPNYTPMHHHKYGMSPPG

GAGMVTPMSVAYGGSGGGGGGAESSSEDLNMYGQSSGEHGGEAAAGQMAFSMSSKKRFRT

KFTTEQKERMMEFAEKLGWRMNKQDEEELKRFCGEIGVKRQVFKVWMHNNKNNARKPPPS

TV

>BraZF-HD12_Bra015898

MKRQMVIKQKSRKSNTSSCTTTSSAISNIRYVECQKNHAANIGGYAIDGCREFMASGNEG

TVEALRCAACGCHRNFHRKEVNTEVVCEYSPPNA

>BraZF-HD13_Bra016538

MKKRQVVIKQRKNSYTTTSSSSNVRYIECQKNHAANIGGYAVDGCREFIASGGEGTDDAL

TCAACRCHRNFHRREVETEVVCEYSPPN

>BraZF-HD14_Bra024382

MDFEDNNNNNNEEEDEMNLHEEEEEEDDAVYDSPPLPPPSRVLKTSTESPDTAGTTSTGG

GGFMVVHGGSSGGGGGSRFRFRECLKNQAVNIGGHAVDGCGEFMPAGIEGTIDALKCAAC

GCHRNFHRKELPYFHHHAPPHQPPPPPPPPGFYRLPAPVSYRPPPSQAPPLQLALPPPPQ

RERSEDRMETSSAEAGGGSGIRKRFRTKFTPEQKERMLALAESIGWRIQRQDDEVIQRFC

QETGVPRQVLKVWLHNNKHTLGKSSSPPLHQHQNPTLPHPPQPSSFHHEQDQP

>BraZF-HD15_Bra025362

MRKRQVVLRRASPEEPSRSSSTASSLTVRGVRYGECQKNHAAAVGGYAVDGCREFMASNG

EEGSVAALTCAACGCHRSFHRREIETEVVCDCNSPPSTGN

>BraZF-HD16_Bra025363

MLEVGAMEMTPKSPEPESETPTRIQPAKPISFSNGIIKRHHHIHHHTLTVTYKECLKNHA

AAIGGHALDGCGEFMPSPSSTPSDPTSLKCAACGCHRNFHRRDPDDSSLASAVPPPSLPP

SSTTAAIEYQPHHRHHPPPPAPPLPRSPTSSSPPPISSSYMLLALSGNNKPGGNSLPFSD

LNFAANNLSTHHHHHNHNHHTPGSRKRFRTKFSQTQKDKMHEFADRIGWKIQKRDEGEVR

DFCRDVGVDKGVLKVWMHNNKNTFNTRRDQPFSGDTTVQKIDNGVAVSGGKNINNNNNNN

NNNNAVDVGVHGGNGLEHDLHGGDGGRFESDSGGAAANGSSSTS

>BraZF-HD17_Bra025644.1

MDMATHTTITSPPKSPEPEPETPNRIQPAKPISFSNGIIKRHHHPTLLFTYKECLKNHAA

ALGSHALDGCGEFMPSPSLVSTDPTSLKCAACGCHRNFHRREPGNDSSIRPPPSSVAATI

EYQPHHRHHPPPPPQLPPPRSPNSSSPPPISSSYMLLSLSGTNNNNNLAFSGNNHHHQTG

SRKRFRTKFSQFQKEKMHEFADRVGWKMQKRDEDDVREFCRQIGVDKSVLKVWMHNNKNN

FNRRDIQFSVAAAGATEIHKTDNGGGGIHAPIPAGETNNNGCNELHHSVSNGGGGFDSDS

GGGGGAHGGDVNGSSSS

>BraZF-HD18_Bra025688

MKKRQVVIKQRKISYTTTTSSSNVRYVECQKNHAANIGGYAVDGCREFMARGGEGTDDAL

TCAACGCHRNFHRREVETEVVCEYSPPN

>BraZF-HD19_Bra026219

MDIASQDDHDMPIPLSTTFVGSGGGHGHMIHHHHDHHASDSAPPTHNHNNITTTQPPQMQ

LHGNGHGNSNDHHHQDPHHIGYNAIIKKPMIKYKECLKNHAAAMGGNATDGCGEFMPSGE

DGSIEALTCSVCNCHRNFHRKEVEGETTATAISSHHQPPPPRKLMVNHHNIRSAMPHQMI

MPIGVSNYRYMHNLESGDFMEQDGVTTASRPPAYNQKKRFRTKFTPEQKEKMLSFAEKVG

WKIQRQEDCVVQRFCEEIGVKRRVLKVWMHNNKLHFSNKNTSNNINLEGNDNDKINNVNN

VDVSGNNDMI

>BraZF-HD20_Bra026589

MEVASQEEHDMPIPINTTHGGHGHMIHRHHDHRHHNPANSTHPNPLMVLSNGNGFGQNHD

DPDHHNVGYNIMISNNNNKEKHVVNKYKECLKNHAASTGGHAIDGCGEFMPSGEEGSIEA

LTCSACNCHRNFHRREIEGEDKTYFSPYHHNQPQRNPMFHHHHKMTKSPLPQQMIMPLGV

ATTTVSNTESEEDLPFQQPPPPYNHGGHNQKKRFRTKFTQEQKEKMLSFAERIGWKMQRQ

EESVVQKFCQEIGVRRRVLKVWIHNNKHNLSKKSNNNNVEISAGNNDINKALAGNLAPSS

>BraZF-HD21_Bra026812

MQSSCLYRECMRNHAAKLGSYAVDGCREYSQPSTGDLCAACGCHRSYHRRIEVQPSGQVT

RARFPFTSLRRVKQLARLKWKAAAEDREEQEEQEEEDTEETSTEERMTVKRRRKSKFTAE

QREAMRDYAAKLGWTLKDKRAVREEISVFCERIGVTRYLFKTWVNNNKKFYH

>BraZF-HD22_Bra026830

MEIASQEDHDMPIPLNTAFVGGGGGHGHMIHHHNDHHAANSAPPTHNNNNITTQPPQMPL

HGNGHGNNHDHRQHQDPHHVSYNAIIKKPIITYKECLKNHAAAMGGNATDGCGEFMPSGE

DGSIEALTCSACNCHRNFHRKEVEGETAATAISSFHQPPPPRKLMLNHHNIRSAMPHKMI

MPVGVSNYRYMHHSESDDFMEEDGVTTASRPPPYNQKKRFRTKFTPEQKEKMLSFAEKVG

WKLQRQEDSVVQRFCEEIGVKRRVLKVWMHNNKLHFSKKDSSNSINLEDNDNEKINNVNN

VDLSGNIDMTKIVP

>BraZF-HD23_Bra027455

MDETKSKIEEKCRRRTKATPICRETGDHVHSPPTRITKSTRPTHAPPPNLESIFRLTPEP

RYGECRKNQAASAGTTAYDGCGEFVSANPGEDSFDCAACGCHRSFHRKESLSDGILEVLK

ISPSQFRQIFCSPYGEAEEKKRIAMDKSPEEEEAARAKRLKTKFTAEQTEKMRSYAEKVG

WKLSSEGRERVREFCDGIGVTRKNLRVWMNNHKEANGRVDEEEEGRVKRFKTKFTAEQTE

RMRSYAEKLRWKVGPEDREETEVFCNEIGVNRNNFMIWMNNHKEGRD

>BraZF-HD24_Bra031051

MKRRQVVIKQRKSSYTTTSSSSNVRYIECQKNHAANIGGYAVDGCREFMASGGEGTDDAL

TCAACGCHRNFHRKEVETEVVCEYSPPS

>BraZF-HD25_Bra031859

MDFEEEINLHEEEEEDALYDSPPLPPPSRVLKASTESPDTAGTTSTGEGGFMVVHGGSSL

GGGGRFRFRECLKNQAVNIGGHAVDGCGEFMPAGIEGTIEALKCAACGCHRNFHRKESLY

FHHHHAPPQQQHHPPPPGFYRLPAPVSYRPPPSQAPPLQLALPQRERSEDRMETSSAEAG

GIRKRFRTKFTAEQKERMLGLAERIGWRIQRQDDEVIQRFCQETGVPRQVLKVWLHNNKH

TLGKSSSPPLHHHQNPTLPPQSSSFHHEQDQP

>BraZF-HD26_Bra036852

THALIANA HOMEOBOX PROTEIN 28); DNA binding / transcription factor

MEHGGKCNAIATTTKISTKILEAKPHTNSYPKAKPGLDPTLPPFLVKKENHKPSSRGDQE

TKYKECQKNHAVSTGGHVVDGCCEFMPGGEEGTLGALKCAACSCHRSFHRKEVYEHINST

QELMSPAFYRSGSLYKAMIQPRGLYLPKKKRIRTKINEEQKEKMKEFAERLGWRILKKDE

EEINKFCRLVNLRRQVFKVWMHNNKQAMKRNGNACNLQK

>BraZF-HD27_Bra037224

MEVREKKDNKIEMERRQSSANQNIQDPRLPPYTYSQTADKEKPTTKRNGSDRDPDLDTNP

TSIAPAPRSYARPQTTSPTRRVSYRECQRNHAASSGGHVVDGWGEFMSSGEEGTAESLLC

AACDCHRSFHRKEVDGMFVVKFNSFGHSQRPLVNRHVSPIMMSFGGGGGGRDPAESSTED

LNRFHQALSGNGVDQFQYHPKKRFRTKFNQEQKERMFEFAEKIGWRMNKSEDEEVNRFCR

EINVKRQVFKVWMHNNKQAAKKKET

>BraZF-HD28_Bra037816

MNLQEEEEDNAVYDSPPLPPSSCVLKASTESPDTAGTNLTGGGGFMVVHGGSRFRFRECL

KNQAVNIGGHAVDGCREFMPAGTEGTIDALKCAACGCHRNFHRKELPYFHHLAPPPPPRG

SYRVPAPVSYRPSPPQASPVQLALPPPAEDRMETSSAEAGGIRKRFRTKFTAEQKERMLG

LAERIGWRIQRQDDEVIQRFCQETGVPRQVLKVWLHNNKHTRGMSSSSPLDQHQNHTLPP

PS

>BraZF-HD29_Bra039038

MDMTLKSPEPEPETPTRIQPAKPISFSNGITKRHHHPIAVTYKECLKNHAAAIGGHALDG

CGEFMPSPSSTPSDPTSLKCAACGCHRNFHRRDPEDSSLTSAVPPPSLPPSSTLEYQPHH

RHHPPPPGPHLPRSPTSSSPPPISSSYMLLALSGTNKPGGNSLPFSDLNFAANNLSTHHL

TPGSRKRFRTKFSQAQKEKMHEFADRIGWKFQKRDEDDVRDFCREVGVDKGVLKVWMHNN

KNTFNNRRDQPVAGGTTVHCVDNGVAVNNAEDDGVHGGNGLEHNLHGDGAEGVNANGSSS

SSGKSNNEA

>BraZF-HD30_Bra039039

MRKRQVVLRRASPEEPSRSSSTASSRMVRGVRYGECQKNHAAAVGGYAVDGCREFMASNG

EEGTVSALTCAACGCHRSFHRRETEVVCDCESPPSTGN

>ZF-HD31 Bra039615

MGDGKTKIISKPSTPDHRIPPYTCSQTTEKENPTTIRNGSNPDPDLDTNPNSFAAPAPRS

YVRPQTTSLTKSVRYKECQRNHAASSGGHVIDGCGAFMSSGKEGTAESLLCAACDFHRSF

HRNKIDGMFVVKFNSFGLSPRPLVSRHVSPVMMSFARGGKDPAESSTEDLNSFINLLVVM

E
