# Supplementary material for: Genome-wide identification and expression analysis of the ZF-HD gene family in pea (Pisum sativum L.)
Source: Front Genet. 2023 Jan 5;13:1089375. doi: 10.3389/fgene.2022.1089375 (PMC9849798; doi:10.3389/fgene.2022.1089375)
Supplement: Supplementary file 5 [file Table4.DOCX]

1.the participating varieties in group 1 were the Kaspa variety and Parafield variety varieties with PsZF-HD gene transcriptome data

kasp ID Flower Immature Pod Immature Seed Nodule Root Root-tip Seedling Stamen Stem Stipule Tendril Pistil

PsZHD13 0.00 0.00 0.00 0.00 0.00 0.00 0.00 0.00 0.00 0.00 0.00 0.00

PsZHD14 0.00 0.00 0.00 0.07 0.00 0.00 0.00 0.00 0.00 0.00 0.00 0.00

PsMIF4 0.00 0.00 0.00 0.00 0.00 0.00 0.00 0.00 0.00 0.00 0.00 0.00

PsMIF1 48.81 88.85 1.97 9.20 4.43 0.00 4.17 128.02 111.83 0.00 30.72 42.29

PsZHD1 10.65 0.00 29.30 2.35 0.00 0.00 61.73 4.29 16.77 19.85 45.05 6.93

PsZHD2 64.29 14.55 27.70 0.68 0.00 0.00 28.47 52.92 2.82 0.09 23.37 172.75

PsZHD3 3.93 0.00 2.12 0.00 0.00 0.00 7.34 10.70 1.21 0.00 0.00 9.67

PsZHD4 7.90 0.00 12.48 0.00 0.00 0.00 12.32 24.25 1.66 10.75 2.87 23.98

PsZHD5 10.33 0.00 32.95 0.00 0.00 0.00 13.09 16.17 6.03 0.62 5.03 12.17

PsZHD6 81.94 36.39 53.76 6.06 0.00 0.00 46.33 49.80 22.00 41.54 61.37 79.01

PsZHD7 15.51 0.00 1.79 1.07 0.00 0.00 5.07 9.89 2.49 4.40 4.30 14.08

PsMIF2 1.00 0.00 0.00 3.61 0.00 0.00 16.59 0.00 6.17 12.01 14.97 0.00

PsZHD8 0.00 0.00 0.00 0.00 0.00 0.00 0.00 0.00 0.00 0.00 0.00 0.00

PsZHD9 5.75 6.86 13.81 0.00 0.00 7.43 40.55 7.12 2.78 6.55 1.12 7.26

PsZHD10 46.80 20.61 35.35 0.10 0.00 0.00 31.62 37.10 10.52 14.13 10.27 64.35

PsMIF3 0.00 0.00 0.00 5.06 66.46 36.07 7.25 0.00 13.89 0.00 3.08 0.57

PsZHD11 0.00 0.00 0.00 0.00 0.00 0.00 0.00 0.00 0.00 0.00 0.00 0.00

PsZHD12 0.00 97.30 11.02 2.59 0.00 0.00 0.00 0.00 0.00 0.06 0.00 0.00

parafield ID flower mmature pod Immature Seed  Nodule root root-tip Seedling leaf stem Stipule Tendril

PsZHD13 0.00 0.00 0.00 0.00 0.00 0.00 0.00 0.00 0.00 0.00 0.00

PsZHD14 0.11 0.00 0.00 0.00 0.00 0.00 0.00 0.00 0.00 0.00 0.00

PsMIF4 0.00 0.00 0.00 0.00 0.00 0.00 0.00 0.00 0.00 0.00 0.00

PsMIF1 44.51 24.46 4.08 1.85 0.00 1.03 3.88 3.86 49.73 0.02 2.20

PsZHD1 12.30 7.11 4.02 4.92 0.00 0.63 49.69 8.73 21.68 18.79 57.98

PsZHD2 35.06 42.51 19.42 0.00 0.00 10.73 44.50 0.00 52.83 0.56 34.02

PsZHD3 3.99 6.07 9.61 0.00 0.00 0.12 0.00 0.00 2.31 0.03 4.08

PsZHD4 11.54 5.40 13.62 0.00 0.00 0.00 0.00 2.21 0.00 1.24 2.12

PsZHD5 32.16 9.94 28.02 0.95 0.00 0.50 13.26 3.28 12.88 0.00 0.00

PsZHD6 69.51 30.21 22.24 12.31 8.35 6.54 30.28 35.96 31.35 30.03 65.19

PsZHD7 14.29 2.39 0.00 0.00 0.00 0.00 0.00 4.18 4.30 1.59 0.00

PsMIF2 0.33 0.00 0.00 5.59 0.00 2.85 40.09 23.59 4.96 3.40 3.22

PsZHD8 0.00 0.00 0.00 0.00 0.00 0.00 0.00 0.00 0.00 0.00 0.00

PsZHD9 3.91 9.49 0.00 0.00 0.00 6.53 0.00 7.11 2.92 7.54 9.10

PsZHD10 29.19 12.25 67.17 1.32 0.00 5.05 18.01 16.37 30.78 6.07 7.38

PsMIF3 7.43 0.00 0.00 7.54 160.37 9.93 0.00 0.00 0.95 1.94 21.94

PsZHD11 0.00 0.00 0.00 0.00 0.00 0.00 0.00 0.00 0.00 0.00 0.00

PsZHD12 1.60 71.63 0.00 0.12 0.00 0.00 0.00 0.00 0.00 0.00 0.00

2. The second group of participating materials was two pea varieties of vegetable peas and grain peas, five periods of post-flowering pod development to determine PsZF-HD gene transcriptome data

gene name CJ1 CJ2 CJ3 CJ4 CJ5 LJ1 LJ2 LJ3 LJ4 L5

PsMIF3 0 0 0 0 0 0 0 0 0 0

PsZHD1 1.743534001 0 0 1.837625236 0 0 0 0 0 1.230664183

PsZHD2 0 1.101346401 0 0 0 2.16970242 0 0 0 0

PsZHD4 0 0 0 0 0 0 0 0 0 0

PsZHD13 0 0 0 0 0 0 0 0 0 0

PsZHD3 0 0 0 0 0 1.118047391 0 0 0 0

PsZHD12 0 2.886351272 0 0 0 3.099109603 0 0 0 0

PsMIF4 0 0 0 0 0 0 0 0 0 0

PsZHD6 0 0 0.652940521 0 0 1.048623657 1.270199226 0 0 1.687609419

PsZHD7 0 1.614925996 0 0 0 0 0 0 0 0

PsZHD11 0 0 0 0 0 0 0 0 0 0

PsMIF1 0 0.672630805 0 0 0 0 0 4.298149348 0 2.16990989

PsMIF2 0 0 0 0 0 0 0 0 0 0

PsZHD8 0 0 0 0 0 0 0 0 0 0

PsZHD9 0 0 0 0 0 0 0 0 0 0

PsZHD14 0 0 0 0 0 0 0 0 0 0

PsZHD10 2.646260955 1.7855799 1.180880026 0 1.474048018 1.229400698 0 0 1.041355741 0

PsZHD5 2.730571468 5.572448176 4.890071925 12.21254984 0 12.54379796 3.304804871 7.924301416 3.833496195 2.955220019

3.The third group of participant materials was two pea varieties with different cold effects, a cold-resistant winter forage variety and a cold-sensitive spring dry pea variety, were subjected to low-temperature treatment at different developmental stages with PsZF-HD gene transcriptome data

gene ID TeN0 TeN1 TeN2 TeL0 TeL1 TeL2 ChN0 ChN1 ChN2 ChL0 ChL1 ChL2

PsMIF3 1.81 2.99 0.33 1.51 1.57 1.29 1.48 2.95 3.72 2.40 1.92 2.60

PsZHD1 8.69 9.69 9.80 11.17 7.69 13.16 7.57 8.88 8.50 8.94 9.34 12.52

PsZHD2 7.53 7.91 5.07 6.53 6.01 5.00 3.29 3.94 4.84 4.92 4.69 3.81

PsZHD4 4.49 4.08 3.38 3.53 2.74 3.02 2.17 2.34 2.92 3.05 2.72 2.38

PsZHD13 0.00 0.00 0.00 0.00 0.00 0.00 0.00 0.00 0.00 0.00 0.00 0.00

PsZHD3 3.87 3.61 2.58 3.91 3.99 3.26 1.87 2.50 2.75 2.39 2.90 1.76

PsZHD12 0.09 0.10 0.20 0.00 0.00 0.00 0.07 0.00 0.00 0.07 0.00 0.00

PsMIF4 0.00 0.00 0.00 0.00 0.00 0.00 0.00 0.00 0.00 0.00 0.00 0.00

PsZHD6 15.00 14.22 14.19 15.58 10.03 12.52 11.32 12.15 11.36 12.89 10.86 10.98

PsZHD7 1.74 1.35 1.81 1.49 1.54 1.19 1.38 1.70 2.01 1.87 1.57 1.00

PsZHD11 0.00 0.00 0.00 0.00 0.00 0.00 0.00 0.00 0.00 0.00 0.00 0.00

PsMIF1 2.46 4.32 4.26 2.87 2.83 5.03 2.54 4.20 3.99 3.03 2.01 3.34

PsMIF2 4.12 5.63 4.20 6.48 2.95 3.24 4.01 4.69 3.76 4.08 2.18 2.54

PsZHD8 0.00 0.00 0.00 0.00 0.00 0.00 0.00 0.00 0.00 0.00 0.00 0.00

PsZHD9 3.15 2.26 2.77 2.22 1.63 1.41 1.78 1.80 2.16 2.28 1.66 1.38

PsZHD14 0.20 0.18 0.08 0.31 0.15 0.29 0.00 0.00 0.00 0.00 0.00 0.00

PsZHD10 15.43 14.58 10.60 12.38 18.62 17.99 10.13 12.36 13.66 12.07 17.00 16.23

PsZHD5 2.41 1.94 1.60 2.10 2.22 2.56 2.12 2.51 2.37 2.49 1.84 1.79

4.expression of PsZF-HD gene of the fourth group of participant materials in different N-treated peas at three developmental stages in different tissues

gene ID RootSys_A_LN RootSys_A_HN Roots_F_LN Roots_B_LN Nodules_A_LN Nodules_B_LN Nodules_G_LN Shoot_A_LN Shoot_A_HN Leaf_B_LN Upperleaf_C_LN Lowerleaf _C_LN Tendrils_BC_LN Stem_BC_LN Peduncle_C_LN Apical node_B_LN Flowers_B_LN Pods_C_LN Seeds-12dap

PsMIF3 5.025661126 3.097686284 2.653260336 1.980588404 0.606648272 1.609786772 1.961977659 0.14636786 0.789053053 5.288882792 6.823048482 0.841742321 3.831444899 4.396820926 4.090674814 0.164423068 0.056672681 0.146465778 0

PsZHD1 0.838343372 0.784008633 0.843312584 0.814574873 1.11316203 1.358096131 0.590742977 11.72473001 13.40521906 9.035477892 7.658332271 9.842480219 6.951208333 7.611142639 2.368315033 24.59425317 2.979876048 13.76214212 3.249621324

PsZHD2 0.299521155 0.290112458 0.836004914 0.072135445 0.220948698 0.156347726 0.103490344 3.61434745 3.362378092 0.030414878 0.032413532 0.110366214 0.370333337 2.337530634 2.324202887 5.713018128 11.97583932 15.26723093 3.376879318

PsZHD4 0.023667118 0.022923676 0.047945465 0.025649512 0.534233051 0.500339132 0.672887026 4.071597846 3.188200849 2.855093911 3.111863328 2.302278996 1.316810259 1.284992642 2.595827993 4.624954258 6.288370001 24.8101153 1.106556556

PsZHD13 0 0 0 0 0 0.03936746 0 0 0 0 0.06529233 0 0 0 0 0 0 0 0

PsZHD3 0 0 0.027793112 0 0 0.020141491 0 3.757909718 3.53190577 0 0.050108067 0.113743393 0.257209322 0.4877235 1.627591219 4.48067953 6.879522359 37.52177163 2.712517396

PsZHD12 0.093718622 0.045387345 0.132900339 0 0 0 0.020816739 0 0 0 0.04563912 0 0.022671272 0 0 0.101183426 0 2.523718028 82.60189172

PsMIF4 0 0 0 0 0 0 0 0 0 0 0 0 0.029345066 0 0 0 0 0 0

PsZHD6 1.220869772 1.336093173 1.149909851 1.873004575 1.705297219 2.104280559 1.394635143 20.40448584 19.42838513 16.44657849 12.26141191 15.65164438 25.23030313 25.01284308 12.82629468 33.1753775 32.18016323 58.49437661 5.693983354

PsZHD7 0.569684413 0.62376173 0.371313187 0.778463444 0.230218908 0.174543763 0.275082719 3.213710358 3.240371671 0.63381953 0.747841495 0.766645216 0.167770082 0.181442966 0.088707621 6.230830069 4.682358984 14.00685813 1.034870881

PsZHD11 0 0 0 0 0 0 0 0 0 0 0 0 0 0 0 0 0 0 0

PsMIF1 0.894314939 0.511858634 0.411756024 1.299641423 0.512776199 0.167102302 0.117381042 6.602690986 4.712569756 0.092877154 0.079184315 0.089872681 38.66618258 91.1832294 105.2957104 110.6649933 40.26895404 0.117285942 19.57237562

PsMIF2 1.731688478 1.041954045 1.583960138 1.023674905 2.752259453 2.619336779 1.456976823 7.640731228 5.131800099 3.237134057 2.683221313 1.21816192 3.300495606 5.099024368 1.491742963 1.614671472 0.097638326 0.403740872 0

PsZHD8 0 0 0 0 0 0 0 0 0 0 0 0 0 0.021409311 0 0 0 0 1.53218092

PsZHD9 0.34530308 0.27364603 0.356121296 0.238143938 0 0.07373681 0.153397136 3.047162417 2.602284004 2.209019636 1.406393862 1.630930752 1.063128561 0.568122214 0.477805482 1.355661438 0.327084746 7.27581607 1.623833458

PsZHD14 0.036048715 0 0 0 0 0 0.032028499 0 0 0 0 0 0 0 0 0.03892004 0 0 0

PsZHD10 0.018857246 0.091324469 0.359094109 0.102183789 0.300466439 0.110737538 0.025131379 11.1278973 10.79611485 11.52936736 17.50300189 14.86269967 6.012341891 2.478351081 5.808060858 9.141302926 15.2697406 35.473499 4.802292578

PsZHD5 0.347091678 0.168094337 1.054721045 0.376164602 1.152180864 1.100663458 0.863473524 1.601223298 1.168919221 0.729580337 2.501596636 1.95679024 2.787611629 3.798941718 2.874619543 7.83202331 3.539070846 5.174075971 0.794149765
